# Supplementary figures and images for: Honeybees adapt to a range of comb cell sizes by merging, tilting, and layering their construction
Source: PLoS Biol. 2025 Aug 26;23(8):e3003253. doi: 10.1371/journal.pbio.3003253 (PMC12380279; doi:10.1371/journal.pbio.3003253)

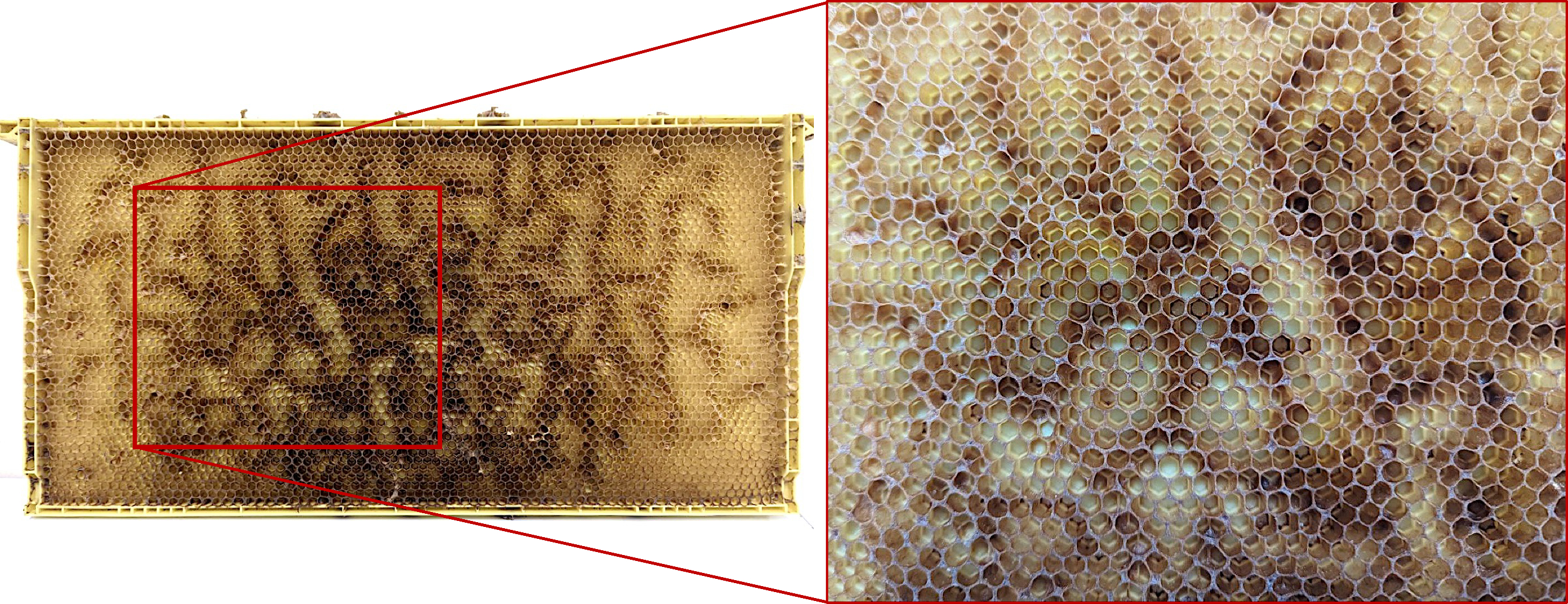

Supplement: S1 Fig — Honeycomb built on one of the commercial frames inside our hives using the merging strategy. A section of comb is magnified to show the occasional combination of the plastic edges on the foundation to build larger cells on top of them. (TIF) [file pbio.3003253.s002.tif]

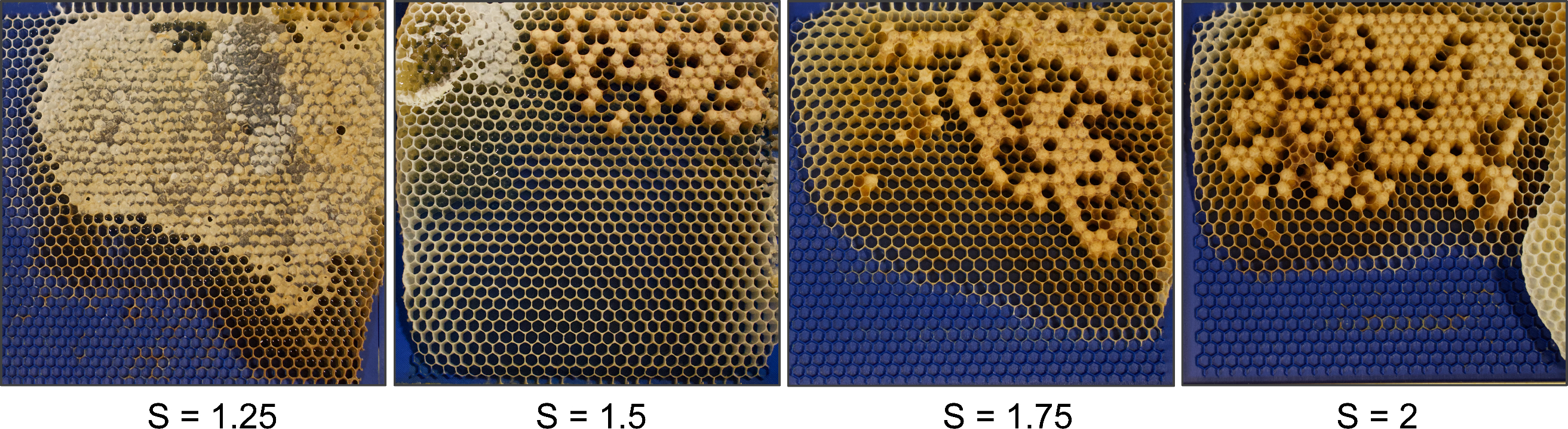

Supplement: S2 Fig — Sample frames with larger cell size foundations are either used for raising drone brood or honey storage. (TIF) [file pbio.3003253.s003.tif]

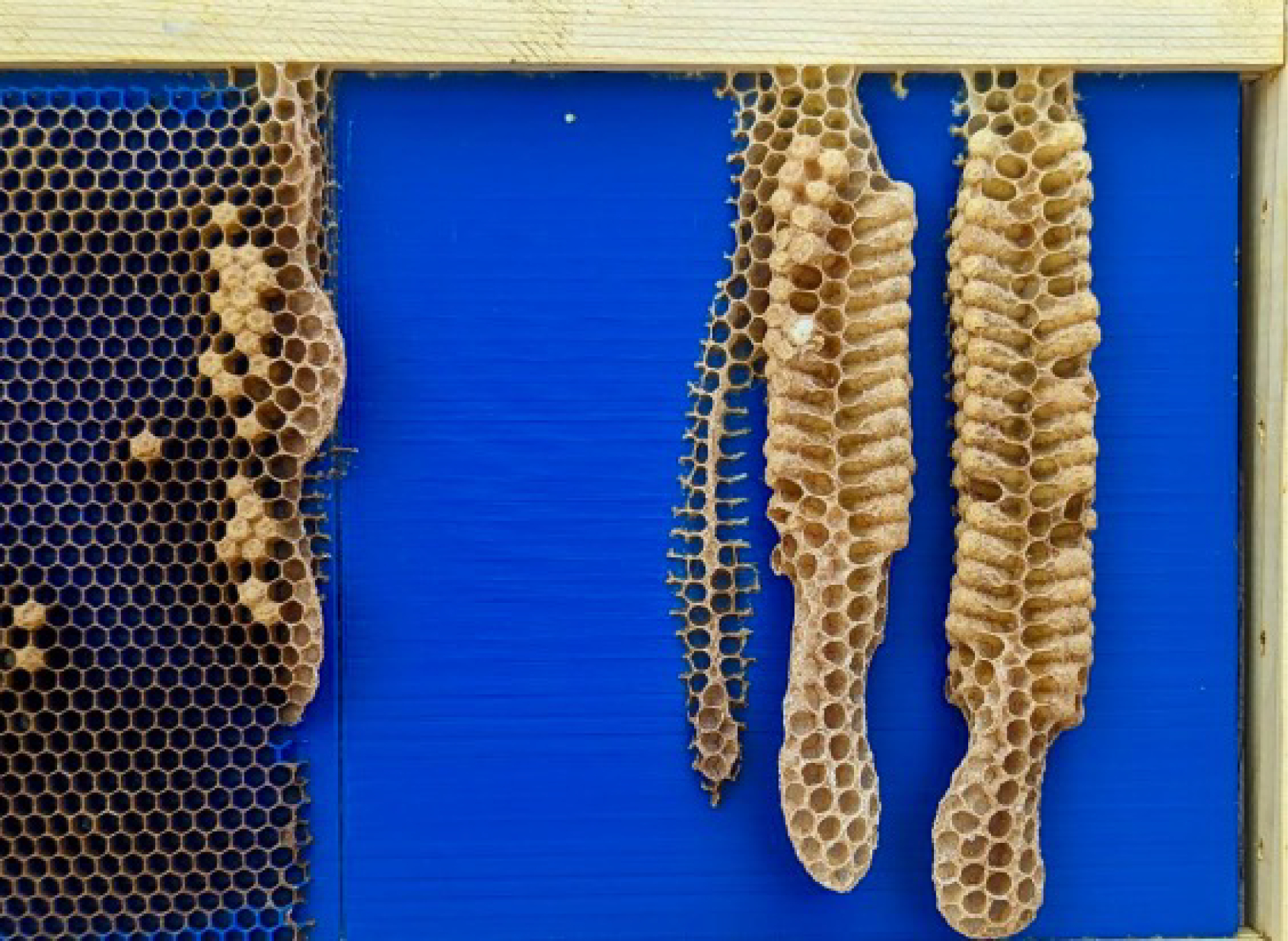

Supplement: S3 Fig — Honeycomb containing drone brood built on the flat side of the experimental frames. This is used to compute the value of 20° as the natural tilt of the drone comb, with a standard deviation of 2.43° and 95% CI: [18.86, 21.14]. (TIF) [file pbio.3003253.s004.tif]

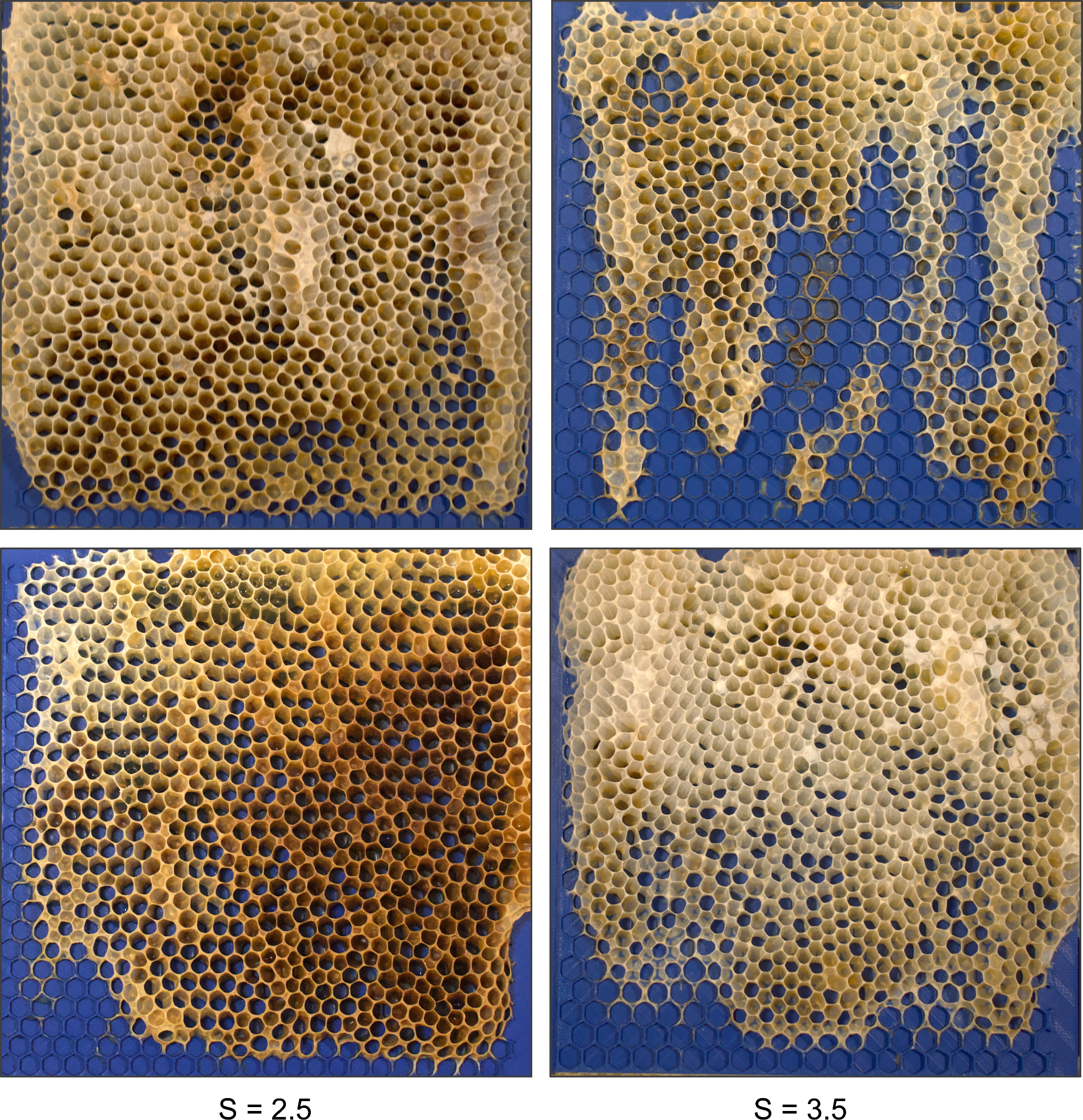

Supplement: S4 Fig — Samples of acquired data on frames with S = 2.5, and S = 3.5 show a combination of tilted or layered modes of building on random sections of the frames. (TIF) [file pbio.3003253.s005.tif]

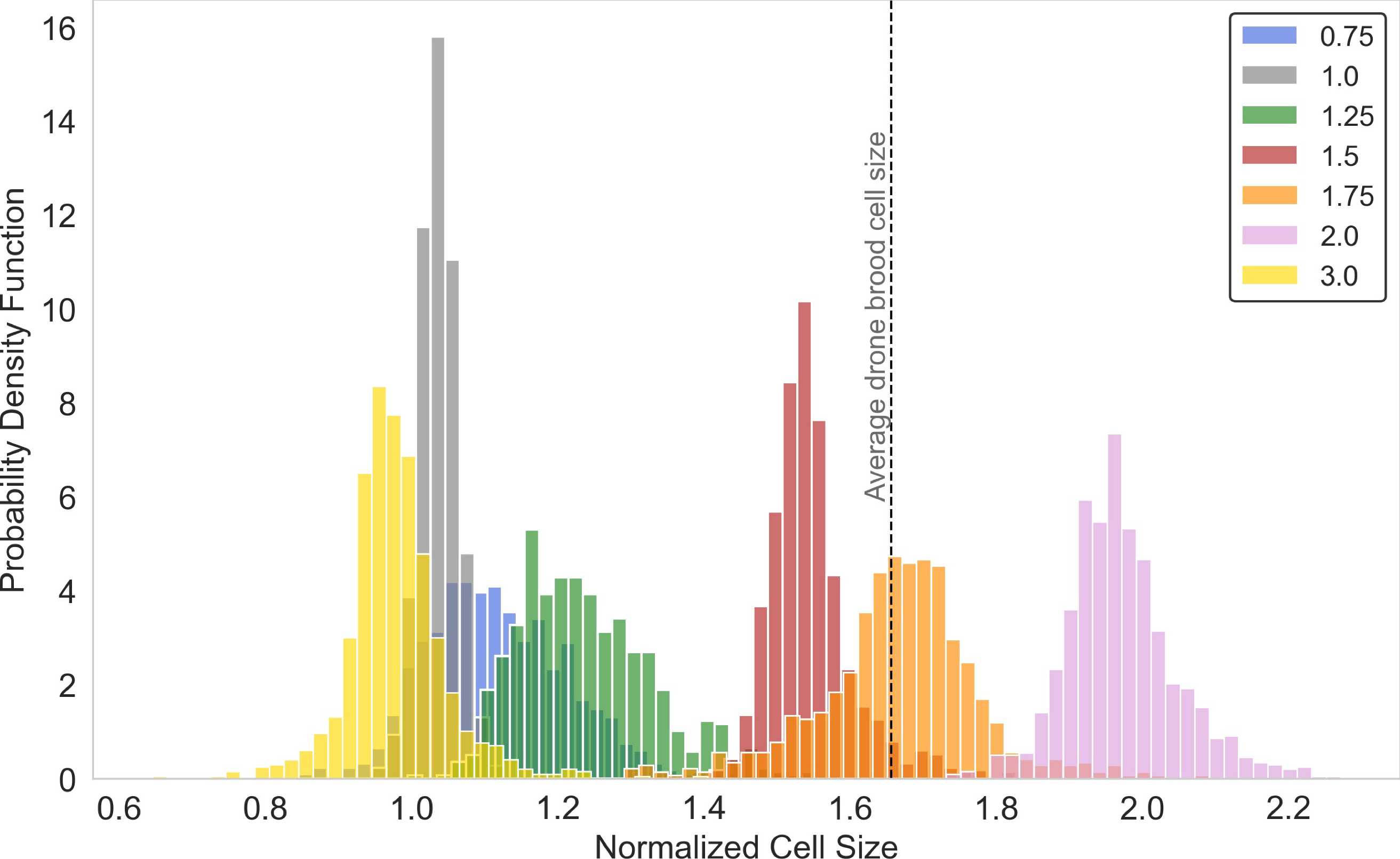

Supplement: S5 Fig — Comparison of cell size distribution of honeycomb built on all of the 3D-printed frames. The given cell sizes (S) are shown with different colors in the figure legend. The data underlying this plot can be found in https://datadryad.org/dataset/doi:10.5061/dryad.z8w9ghxmw. (TIF) [file pbio.3003253.s006.tif]
