# Supplementary material for: Honeybees adapt to a range of comb cell sizes by merging, tilting, and layering their construction
Source: PLoS Biol. 2025 Aug 26;23(8):e3003253. doi: 10.1371/journal.pbio.3003253 (PMC12380279; doi:10.1371/journal.pbio.3003253)
Supplement: Attachment — Submitted filename: PLOS - Response to Reviewers with diff.pdf [file pbio.3003253.s014.pdf]

Response to Reviewers' comments for the manuscript
(PBIOLOGY-D-24-01668R1)

"Adaptive Cell Size, Merging, Tilting, and Layering in Honeybee Comb Construction"

Golnar Gharooni-Fard, Chethan Kavaraganahalli Prasanna,
Orit Peleg, and Francisco López Jiménez

We believe we have addressed all comments from the reviewers, in addition to other small changes to simplify notation and fix typos. Please refer to the "diff version" appended at the end of this document for a detailed account of all the changes, corrections, and additions to the manuscript. The original text that was modified/deleted is struck-through (in red), and the revised/new text is underlined (in blue).

Response to Reviewer #1

Reviewer #1: Overall, I felt this study was very original and offers a nice complement to the authors earlier PNAS article (Gharooni-Fard et al. PNAS 2022). The study presents novel findings on the adaptability of honeybees in constructing combs with varying cell sizes, and does so using a set of innovative techniques, such as 3D printing and X-ray Microscopy (XRM). This research is highly relevant and significant both for biologists interested in self organisation and collective behaviour as well as for engineers interested in bio-inspired designs. The manuscript is well-organized and clearly written, and makes complex experimental setups and results accessible to non-specialists. The methodological section is also sufficiently detailed to allow results to be reproduced by others. Overall, I think the results are of sufficient general interest to be able to recommend publication in PloS Biology.

We thank the reviewers for carefully reading the paper, identifying the novelty of the work, and for the positive feedback. We will address each of the questions and comments below.

Abstract, introduction & discussion: I think the abstract, introduction and discussion would benefit from being pitched at a more generic level, e.g. also emphasizing the broader implications of the study in the field of collective behaviour and self organisation. For example, a discussion similar to that of Gallo & Chittka (PNAS, 2021, "Stigmergy versus behavioral flexibility and planning in honeybee comb construction") could be nice, along with a more broadly pitched introduction and discussion. My main concern is that the article should be placed better within the larger body of prior work on collective behaviour and self organisation. Even for the specific topic of this article, I think some extra articles could be worth citing: aside from the Gallo & Chittka PNAS commentary, perhaps also Gallo et al. J. Comp. Phys. A (2023), Krishna et al. Sci. Rep. (2022), Smith et al. PloS Biol. (2022) and Yang et al. PloS One (2022).

The focus of our experiments was to rationalize the strategies and modes of building that are employed when bees are faced with foundations having various cell sizes. However, we thank the reviewer for their suggestions, and we agree that reviewing a broader previous work regarding honeybee comb building will give a better understanding of the problem we are addressing in the paper. So, we added the suggested papers to our references.

To provide a broader perspective we made the following changes to the “Introduction”. First, we have included mentions and references to previous work on comb building in honeybee, including other bee species and social wasps:

> *“Previous studies across various honeybee species have shown the precise design of their nest, optimized to maximize storage capacity and brood space [10,17,18]. These adaptations occur as the needs of the colony fluctuate, requiring more or less space for the rearing of the brood or the storage of honey over time [12]. It is worth noting that honeybees are not the only species that adjust their nest structure based on external changes in the environment and colony requirements. Social wasps have also been shown to use architectural solutions to build their nests, similar to honeybees [19,20]. The combs of paper wasps exhibit remarkable adaptability, with structural variations influenced by factors such as nest maturation, overall size, and cell arrangement [19,21,22].”*

To clarify the contributions and broader implications of our work, we added a statement that shows the potential value of accounting for the three-dimensional structure of the comb with a mention and reference of a recent study that visualizes the process of nest building in 3D.

> *“While the specific behavioral rules of individual bees remain unclear, revealing the 3D structure of the comb built under various initial conditions will offer a crucial first step in linking the local behavior to the global building patterns. The 3D progression of the nest, shown in a recent study by Marting et al. [23], reveals that bees prioritize building a well-connected nest of parallel combs that remains structurally and functionally robust, even when faced with experimental disruptions. These findings show the importance of accounting for 3D comb structure and provide valuable insights into the adaptive and resilient nature of the superorganism.”*

We also emphasized the goal of our study in a later paragraph:

> *“By exploring the responses of honeybees to various initial conditions (using foundations with different cell sizes), our objective is to exhibit the range of construction modes bees use to build their nest, as well as the complexity of these structures and their sensitivity to initial conditions.”*

We also made several changes to the Discussion section, in particular the addition of this paragraph:

> *“The flexibility of comb building behavior in bees has been previously discussed as a result of stigmergy [33] combined with a highly rich behavioral repertoire owned by bees [34]. Our experimental results extend our understanding of honeybees' remarkable comb-building adaptability, providing detailed evidence of their diverse construction techniques when faced with varying foundation cell sizes. Our findings align with and strengthen previous research by introducing*

more variety of construction modes in response to specific environmental conditions. The observed variety of solutions might suggest cognitive planning [35,36]--where bees potentially maintain a mental template of the desired outcome and select appropriate building modes based on foundation geometry--our study primarily focuses on how bees demonstrate multiple effective strategies for managing geometric challenges related to cell size, while maintaining structural functionality. However, the cognitive aspects of forward planning at the colony level would require dedicated experiments that are beyond the scope of this paper."

Methods & results: While the methodology is rigorous, the results are mainly described in purely descriptive terms and do not entail any detailed statistical methods. Some of the results on the presented frequencies of the different types of cells and patterns could benefit from a formal statistical analysis though, e.g. using linear mixed models (LMMs) or generalized linear mixed models (GLMMs) with the use of appropriate error structures (e.g. gaussian, binomial or multinomial) and the inclusion of random intercepts to account for colony-specific variation and/or overdispersion (through the inclusion of an observation-level random effect) (both colony-level effects and any overdispersion as well as the specific distribution of the data are currently all ignored). Additionally, including confidence intervals and effect sizes in the analysis would strengthen the statistical robustness of the findings. These analyses could be done e.g. using the lme4 and emmeans packages (for binomial GLMMs and Gaussian LMMs) or using mclogit::mblogit for multinomial GLMMs.

We thank the reviewer for their suggestions. We added a more precise statistical analysis of the results to the paper. The details of all the changes we made are described below.

Lines 13-14: The sentence "For smaller cell size, bees occasionally merge cells to compensate for the reduced space" should be rephrased for clarity. Suggestion: "For smaller cell sizes, bees occasionally merge adjacent cells to optimize space utilization."

Since we do not want to make assumptions about what is "optimized" from the bees' perspective, we rephrased the sentence based on the reviewer 's suggestion to be "*For smaller foundations, bees occasionally merge adjacent cells to compensate for the reduced space.*"

Lines 45-47: Provide more details or examples of how the degree of adaptability and variability in honeycomb structure was previously assessed in 2D studies. Mention specific studies and their findings.

We provided more details about the comb structure analysis in 2D and their findings in the following paragraph in lines 55-72, in the study done by H. R. Hepburn.

Line 64: Clarify the specific contributions of Franklin et al. (2022) to set the context for the current study. For example, "Franklin et al. (2022) used XRM

technology to describe the step-by-step process of honeycomb construction in natural settings, highlighting the intricate structure of honeycomb cells."

We added this sentence to expand on the findings of the study by Franklin et.al:

> *"Their findings contribute to understanding the nest construction mechanism by showing how individual cells grow directly from the hexagonal pattern on the comb's spine, enhancing structural stability through staggered lattice patterns and strategic material deposition."*

Lines 170-172: Provide some extra literature citations supporting the fact that larger cells tend to be used for raising the larger drones or storing honey.

We added four references from the previous findings that support our observations and edited that sentence to reflect those.

> *"According to our observations (which are consistent with previous findings [13,25-27], these larger cells (built on foundations with $1 < S \leq 2$) are not suitable for raising workers, which are normally raised in cells built with $S \approx 1$. Therefore, these larger cells are either used for raising drones or storing honey. See Fig. S4 for some examples of the usages of these cells in our experimental frames. For context, the average drone cell size [28] when normalized is equal to S=1.61."*

Please use a proper statistical model (e.g. a binomial GLMM with colony included as a random intercept fir using glmer::lme4) to give 95% confidence intervals on that 25% proportion. You could also use the emmeans package to test that the observed ratio does not differ significantly from the expected 25%.

We calculate the ratio of covered cells using three technical replicates (i.e., three different S=0.75 experimental frames) with 272, 338 and 480 total cells respectively. We calculated the proportion of covered cells and the 95% confidence intervals (using normal approximation for the binomial proportion since sample size n >> 30). We believe that this is sufficient to demonstrate the uncertainty in the proportion and whether it is significantly different from 25% or not. The proportions and their 95% CIs for each technical replicate and the pooled data is noted below.

- Technical replicate 1:
    - Proportion = 0.239
    - CI: [0.188, 0.289]
    - Z-statistic: -0.426
    - P-value: 0.669
- Technical replicate 2:
    - Proportion = 0.243
    - CI: [0.196, 0.288]
    - Z-statistic: -0.317
    - P-value: 0.75
- Technical replicate 3:

- Proportion = 0.239
- CI: [0.201, 0.278]
- Z-statistic: -0.535
- P-value: 0.593

- Overall:
  - Proportion = 0.240
  - CI: [0.215, 0.266]
  - Z-statistic: -0.744
  - P-value: 0.457

Our null hypothesis: to build a normally sized honeycomb from the S=0.75 foundation, 25% of the cells must be covered to compensate for 25% less space available per cell. Using the proportion h_0=0.25 as our null hypothesis, each individual technical replicate, along with the pooled data, fails to reject the null hypothesis, under a one-sample two-tailed z-test at a significance level of 0.05 (z-statistic along with p-value is noted above), which suggests that the null hypothesis is a plausible explanation. Moreover, since we cannot reject the null hypothesis in each individual technical replicate and in the pooled data, it suggests that the null hypothesis behavior is observed consistently.

Using this result, we modify the write-up about this as follows:

> *"If we hypothesize that bees compensate for the 25% reduction in space per cell (relative to their average worker cell size) by combining cells in our 3D-printed foundations with S=0.75, we would expect them to cover 25% (h_0=0.25) of the cells in the printed foundation to build a hexagonal lattice with S ≈ 1. Interestingly, all individual experimental samples, as well as the pooled data, fail to reject our null hypothesis (h_0 = 0.25) under a two-tailed one-sample z-test for proportions at a significance level α=0.05 (when pooled together: h_{pooled} = 0.24, z = -0.744, P=0.457). For details about the individual replicates, see Table S2 in the Supplementary Materials section. This suggests that bees employ the merging strategy to effectively transform a slightly smaller hexagonal grid (S=0.75) into their commonly used size (S=1), and the behavior has been consistent across our samples."*

> **Again, use a proper statistical model to describe these results (e.g. a gls generalised least squares model with an error structure that allows for differences in variability across both groups, and use emmeans to report effect sizes and 95% confidence intervals). You could also compare the AIC of a gls model with identical or heterogeneous variances to show that a model with different variances fits better.**

As a result of our statistical analysis, here's how we have updated our write-up along with the accompanying figure.

- We have quantified the number of technical replicates in our data and quantified the sample sizes of each.
- We provide the mean estimates with confidence intervals, while in the figure we show mean and standard deviation of the cell-size distribution. The gray dot within each swarm

indicates the mean normalized cell size for that foundation size with the error bars showing the standard deviation.

- We have quantified how cell-sizes of cells built on S=0.75 frames are closer to the reference worker cells than the printed cells on the frame, illustrating this in the figure as well. We quantify the cell size difference between samples using the difference between the mean normalized cell size across samples, as illustrated in the figure below.
- We also quantify the differences of this cell size distribution from that of the worker cell size distribution using a one-tailed two-sample Welch's t-test for the mean (this test accounts for different variances across the samples being compared) and the Levene test to compare variances. We use the Levene test because our data deviates significantly from a normal distribution, breaking the assumptions of an F-test.

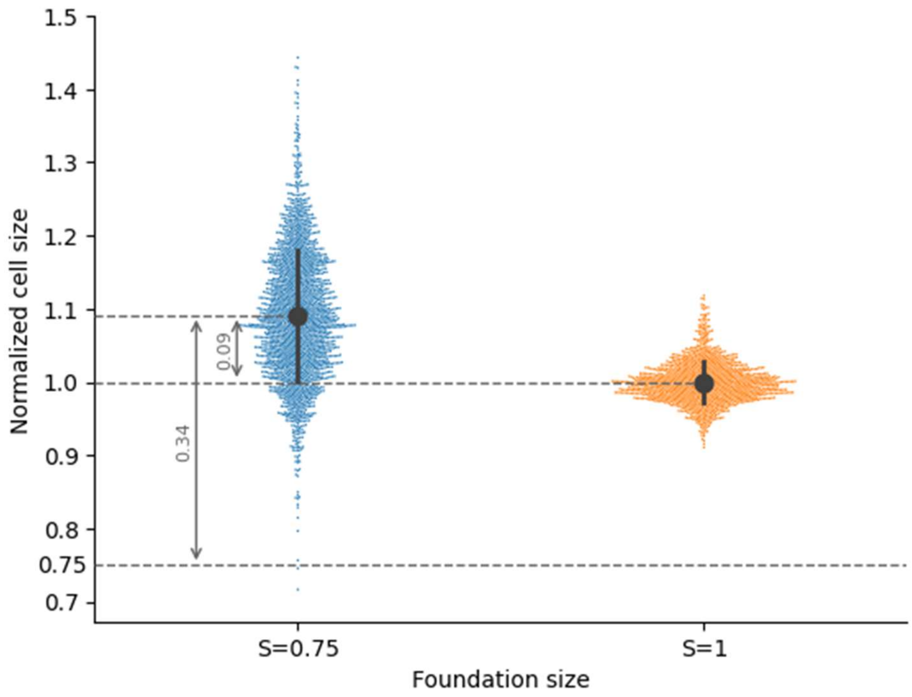

The updated write-up can be found below:

"Fig. 2 K depicts the distribution of normalized cell size for cells built on frames with S=0.75 in blue, and the same for reference cells (S=1) in orange. The normalized cell size is computed by dividing the built cell areas by the mean reference cell area. For better interpretability, we will use normalized cell sizes in future discussions, unless otherwise stated. As depicted in Fig. 2 K, the mean normalized cell size built on foundations with S=0.75 is 0.09 units larger than the mean reference cell size, but 0.34 units larger than the area of the corresponding printed cells S=0.75. This suggests that on foundations with S=0.75, bees are building honeycomb similar to the typical cell size that is closer to the reference cells than to those on the printed foundation. Moreover, apart from being slightly larger than the reference cell size ($\mu_{S=0.75}$=1.09, $\mu_{S=1}$=1, t=44.0385, $P < 10^{-5}$ for a one-tailed two-sample Welch's unequal variances t-test), the variation in cell size for these cells is considerably wider ($\sigma_{S=0.75}$=0.09, $\sigma_{S=1}$=0.03, W=971.173, $P < 10^{-5}$

for the Levene test to compare variances), suggesting less consistency in the cell sizes of the comb built on the smaller foundations. The higher standard deviation could be attributed to the discrepancy between the printed and desired cell sizes and the inherent randomness of the merging process. See Table S1 in the Supplementary Materials section for more details on the number of samples in each category, the individual sample size and statistics."

Lines 174-175: Again use proper statistical methods here as opposed to just describing the results.

We have made the following changes:

- We have redesigned Fig. 3C to show the cell-size trends better. The figure shows the normalized cell size distribution of cells built on each foundation size 1 <= S <= 2. The gray dot within each swarm indicates the mean normalized cell size for that foundation size with the error bars showing the standard deviation. A best-fit linear regression line is illustrated in the figure as well.
- We have statistically quantified terms we have used like "increasing trend" and a "strong positive relationship" between the foundation size and the normalized cell size. The increasing trend has been quantified by fitting a linear least-squares regression line to this data and observing that the slope is statistically significant and positive.
- The correlation coefficient of the data is high (0.97), indicating a strong positive relationship between the foundation size and the built cell size.
- The detailed statistics (mean cell size, std. Dev. And 95% CIs) are shown in Table 1.

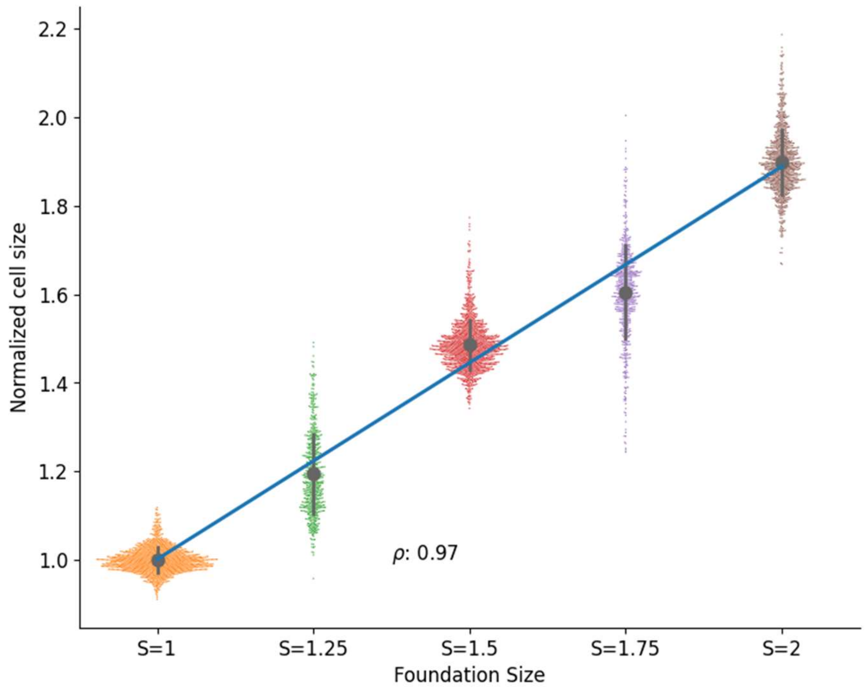

The write-up on the cell-size trends is revised as follows:

> *"The distribution of the size of the cells built on frames with foundations with S=1, 1.5, 2 is shown in Fig 3C, with their detailed statistics shown in Table 1. A linear least-squares regression line that fits the cell size data, using normalized cell size as the response and the foundation size as the predictor, has a positive slope that is statistically significant (β=0.89, $P<10^{-5}$), demonstrating an increasing trend of built cell sizes with the foundation size. Moreover, Fig. 3 C suggests that this increasing trend in cell sizes is fairly monotonic, with the strength of the trend highlighted by a high correlation coefficient (Pearson's correlation coefficient $\$\rho$=0.97, $P < 10^{-5}$), indicating a strong positive relationship between the foundation and the final cell size. This provides strong evidence of the bees' ability to effectively utilize the printed foundations by adjusting honeycomb cell shapes and sizes in response to structural changes. According to our observations (which are consistent with previous findings [14, 25-27], these larger cells (built on foundations with $1<S \leq 2$) are not suitable for raising workers, which are normally raised in cells built with $S \approx 1$. Therefore, these larger cells are either used for raising drones or storing honey. See Fig. S4 for some examples of the usages of these cells in our experimental frames. For context, the average drone cell size [28] when normalized is equal to S=1.61."*

> **Lines 183-184, lines 187-189: Fit proper statistical models to these data (e.g. a linear mixed model or generalised linear mixed model) and report effect sizes and 95% confidence intervals using emmeans. Nonlinearity could be included via a natural cubic spline (ns(), e.g. with df=2) term in the model.**

We have made the following changes:

- We use linear regression to show the positive trend of angle of tilt with the foundation size.
- Moreover, we use a one-tailed t-test between the angle of tilt for S=1.5 and S=1.75 to show that S=1.5 deviates from this trend by being more tilted than S=1.75, indicating that this linear trend is not monotonic. Below is the accompanying figure showing the distribution of angle of tilts for each foundation size and the linear least-squares regression line fit to the data.
- The detailed statistics (mean cell size, std. Dev. And 95% CIs) are shown in Table 1.

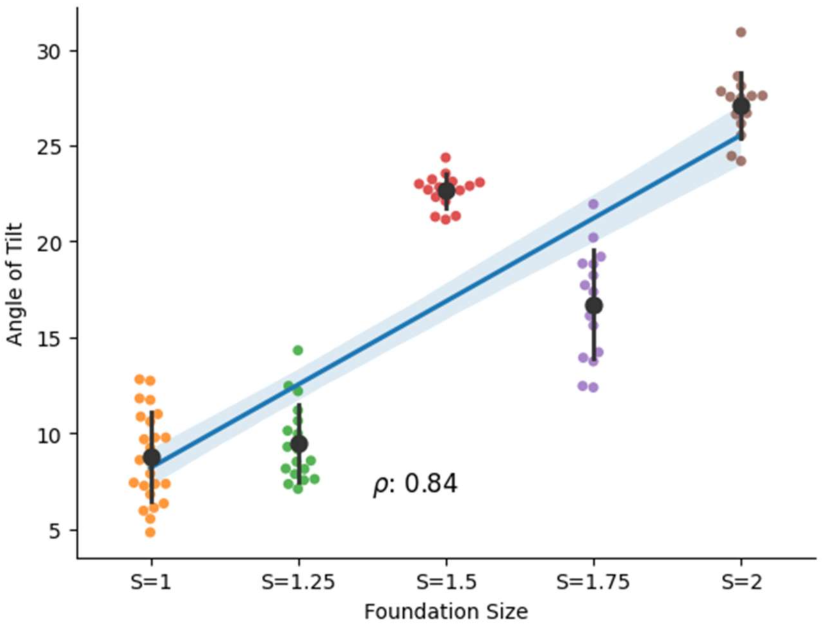

We have updated the write-up describing these results as follows:

> *"In our experiments, we note that cells on the control frames with S=1 are tilted, on average, by 8.74º against the direction of gravity. For comparison, we computed the average tilt angle of natural drone cells to be 20º (see Fig S5 for more information). The tilt of the drone comb is measured manually using images of natural drone comb built on a flat surface, such as Fig. S5. The mean angle of tilts, their 95% confidence intervals and standard deviations for each foundation size 1 ≤ S ≤ 2 are shown in Table 1.*
>
> *Fig 3D shows the tilt distribution of the comb cells built on various foundation sizes, along with a best-fit linear regression line. It is evident that there is a gradual increase in cell tilts with increasing values of S, with a linear least-squares regression line fit to the angle data (response: angle of tilt, predictor: foundation size) showing a statistically significant positive slope (β = 17.36, $P < 10^{-5}$). Although the linear positive trend is strong (Pearson's correlation coefficient: ρ=0.84, $P<10^{-5}$), this relationship is not strictly monotonic. In particular, cells built on foundations with S=1.75 are, on average, less tilted compared to those with S=1.5 (under a one-tailed two-sample Welch's unequal variances t-test, t=-8.04, $P<10^{-5}$). This trend suggests that cell size is not the only influential parameter and the tilt may be influenced by various structural factors of the hive, such as cell position on the frame, usage, content, etc."*

> **Lines 222-224: Fit proper statistical models (e.g. a linear mixed model or generalised linear mixed model) to these data and report effect sizes and 95% confidence intervals using emmeans.**

This is how we are reporting the results after conducting more stringent statistical testing:

- We have quantified the number of technical replicates in our data and quantified the sample sizes of each.
- We provide the mean estimates with confidence intervals, while in the figure we show mean and standard deviation of the cell-size distribution. The gray dot within each swarm indicates the mean normalized cell size for that foundation size with the error bars showing the standard deviation.
- We have quantified how cell-sizes of cells built on S=3 frames are closer to the reference worker cells than the printed cells on the frame, illustrating this in the figure as well. We quantify the cell size difference between samples using the difference between the mean normalized cell size across samples, as illustrated in the figure below.
- We also quantify the differences of this cell size distribution from that of the worker cell size distribution using a one-tailed two-sample Welch's t-test for the mean and the Levene test to compare variances. We use the Levene test because our data deviates significantly from a normal distribution, breaking the assumptions of an F-test.

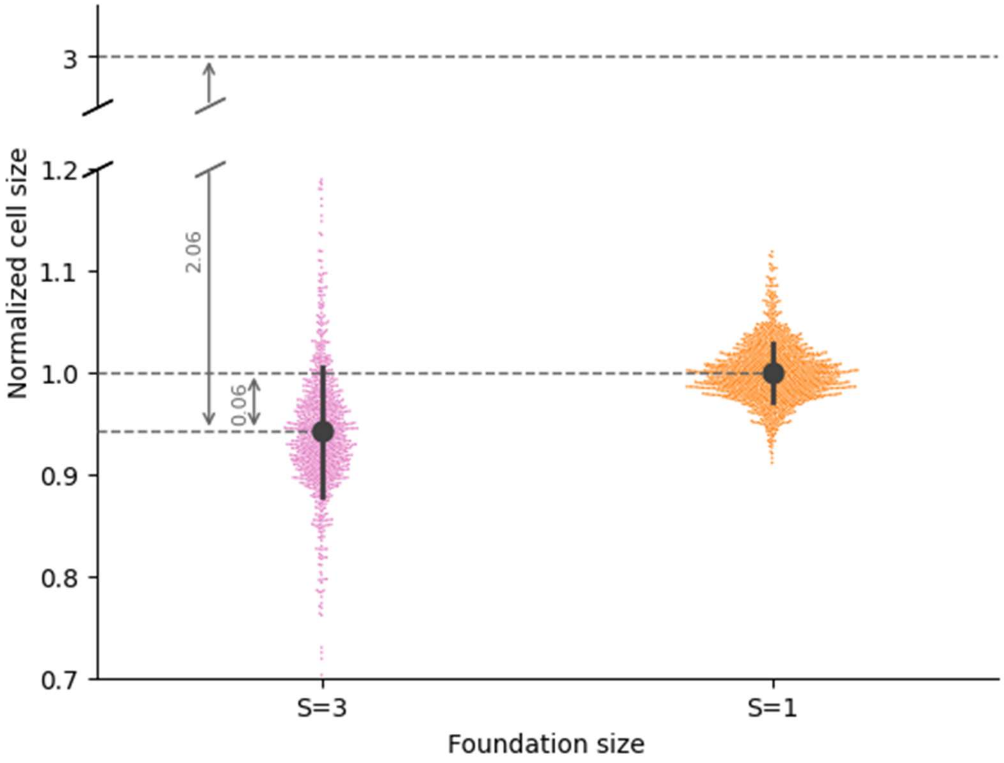

We have modified the write-up of this result to include the statistical details as follows:

> *"Fig. 4 D depicts the distribution of normalized cell sizes of cells built on foundations with S=3 and the reference cells (S=1), with their means and standard deviations included. We find that the mean normalized cell size of cells built on foundations with S=3 is 0.06 units smaller than the mean of the normalized reference cells, whereas these cells are 2.06 units smaller than the printed cells in the S=3 frames, showing that cells built on foundations with S=3 are much closer in size to reference worker cells than to their printed foundations. Typically, these*

cells are smaller than reference worker cells ($\mu_{S=3}$=0.94, $\mu_{S=1}$=1, $t = -26.67$, $P < 10^{-5}$ for a one-tailed two-sample Welch's unequal variances t-test), while the variation in their normalized cell sizes is wider than that of the reference cell size distribution ($\sigma_{S=3}$=0.06, $\sigma_{S=1}$=0.03, W=285.59, $P < 10_{-5}$ for the Levene test to compare variances). The similarity in size of the cells built on S=3 foundations to that of the reference cells suggests that the two-layer strategy employed by bees effectively transforms the printed foundation of S=3, which is not suitable for any purpose in the hive, to S=1, which is the most commonly used."

Response to Reviewer #2

The authors report interesting results regarding the way bees reconcile, in their adaptive hive building, suboptimal foundational geometry imposed upon them. I found no issues with the experiments, methodologies, and analyses conducted to produce the results. This study appears to be of interest to a rather wide audience and suitable for the scope of PLOS Biology. The paper is fairly well written, although I think the authors could make the writing slightly more consistent (in terms of terminology used) and cohesive --- which would assist the reader's understanding of the main findings in this short report. Specifically, I would like the authors to address the following minor comments:

We thank the reviewer for their positive feedback, the careful reading of our manuscript, and for making numerous useful suggestions. We will address each specific comment below.

Lines 37-38: Instead of "...differing combination of cell sizes, alignments, etc. at the boundaries", I suggest <differing geometries of the cells at the boundaries (notably in terms of size and alignment)>.

We edited the text to match the reviewer's suggestion.

Line 74: "...derived from the value of the diagonal..." --- <length> seems to be a better word here.

We edited the text to match the reviewer's suggestion.

Lines 74-75: "...perfect hexagon..." --- <regular> hexagon? (keep it consistent throughout the paper).

We agree with the reviewer's suggestion and replaced "perfect" with "regular" across the paper.

Line 96: "...overall structure of the comb in each category." --- It appears you are using both "modes" and "categories" to refer to the same thing in the same sentence. Then in the next sentence you use categories. Better keep it consistent and call it, say, <construction modes> from the beginning.

We agree with the reviewer and edited the text to consistently use the phrase "construction mode" across the paper.

Lines 102-103: "...faced with smaller cell sizes..." --- <foundational> cell

sizes? There should be a clear distinction throughout the text between the 3D-printed cells provided as foundation and the wax cells built by the bees. Using just "cell" for both of them is confusing.

We agree with the reviewer and double-checked the full document for instances of this to clarify the distinction of “printed” vs. “built” cells.

Lines 123: "...axis" ---> <axes>

We thank the reviewer for catching the typo, that has been fixed.

Lines 125-126: "...adjacent plastic edges beneath the cells constructed on top of them." --- This sentence is somewhat convoluted. I recommend rephrasing it to increase clarity.

We rephrased this sentence for more clarity: *“For example, the XZ slice in Fig. 2 F shows how the comb material is used to join adjacent plastic edges beneath the cells”.*

Line 130: "The distribution of covered cells..." --- Again here, it will be helpful for the reader to consistently distinguish in the text between "foundational"\"printed"\"scaffold" cells and the cells built by the bees.

We agree with the reviewer and have added the word “**printed**” here to be consistent and clear.

Lines 142-146: While you provided a reasonable explanation for the higher standard deviation, an explanation for the clearly higher average ($S = 1.12$ vs. $S = 1.04$) is missing. Could this be explained?

As discussed in the response to Reviewer 1, we have made significant changes to the discussion of these results. However, we do not have a good explanation to the differences in average. In particular, we do not know if they are limitations in our experiments (e.g., limited data or edge effects due to not having an infinite lattice) or a significant different. We agree with the reviewer that it is an interesting feature, and we are planning to conduct further experiments, including other cases in the range $0.5 < S < 0.75$, to obtain further data and explore this phenomenon.

Line 150: Does "worker-size foundations" mean $S = 1$? Better make this clear.

Yes, that is what it means, and we edited the text to clarify it by adding this parenthetical phrase to the text: *“(equivalent to our 3D-printed frames with S=1)”*.

Lines 163-165: "...Fig. 3B reveals a consistent behavior: bees construct honeycomb cells along the edges of plastic foundations, regardless of the

increasing cell sizes observed in the samples." --- The purpose of this statement, and its relation to the next statement, is unclear.

We rephrased this sentence for more clarity.

"...Fig. 3B reveals a consistent behavior: bees follow the printed foundations, regardless of increasing cell sizes."

Line 168: "...a clear positive correlation between the foundation and the final cell size." --- What's the correlation coefficient?

We thank the reviewer for the suggestion. The correlation coefficient is 0.97.

Lines 170-172 According to our observations, these larger cells that are too large for raising workers, are either used for raising drones or storing honey (see Fig. S4 for some examples)." --- From the distributions in Fig. 3C, it seems that S = 1 lies outside the distribution of the cells built by the bees for all the foundations with S > 1. Namely, it appears that all cells in these cases are "too large" for raising workers. Are workers not raised in these hives at all? Could you make this statement clearer in these regards?

Our experimental frames are scattered in the hives along with several normal frames, usually in a 50-50 ratio. So, while it is true that the workers are not raised in the larger cells (S>=1.5), the hives still have plenty of normal-sized frames (commercial frames with S=1) which they'll use for raising workers. We have explained this more carefully in the text, when describing our methods. We also redesigned Fig. 3C and quantified these descriptive statements for more clarity.

Lines 174-175: "While the dashed line aligns well with the S = 1.75 and closely follows the S = 1.5 distributions, it is quite far from S = 2." --- I think it is more accurate to say <While the dashed line matches the mean of the S = 1.75 distribution ({provide the mean}) and overlaps with the S = 1.5 distribution, it does not overlap with the S = 2 distribution.>

We revised this sentence to match the reviewer's suggestion.

Line 187: "...this relationship is not strictly linear." --- <monotonic> seems to be more accurate than linear here.

We agree that monotonic seems to be a more appropriate term to use here. We have modified the text as follows:

Although the linear positive trend is strong (Pearson's correlation coefficient: ρ=0.84, $P<10^{-5}$), this relationship is not strictly monotonic. In particular, cells built on foundations with S=1.75 are, on average, less tilted compared to those with S=1.5 (under a one-tailed two-sample Welch's unequal variances t-test, t=-8.04, $P<10^{-5}$). This trend suggests that cell size is not the only influential parameter and the tilt may be influenced by various structural factors of the hive, such as cell position on the frame, usage, or content.

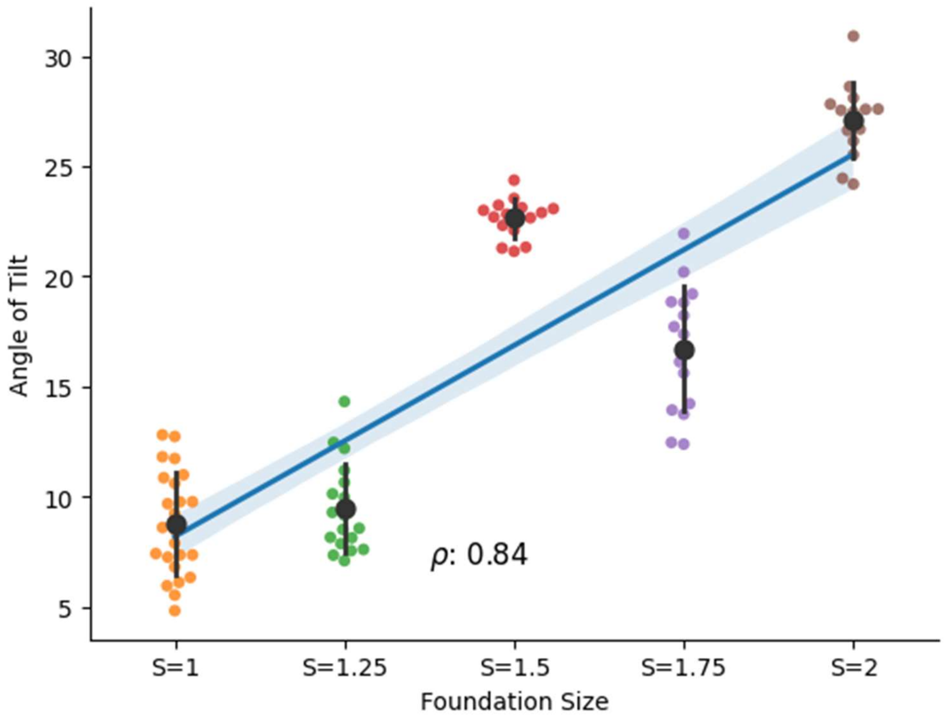

Lines 187-188: "Notably, cells built on foundations with S = 1.75 are, on average, smaller than those with S = 1.5..." --- The numbers in Table 1 indicate the opposite - the cells built on S = 1.75 are larger than those built on S = 1.5 (S = 1.67 vs. S = 1.55 on average, respectively). Did you mean that they have <smaller average tilts>?

Yes, they do. We thank the reviewer for pointing out this typo. We corrected the sentence. Please refer to the response to the previous question for more information.

> *"In particular, cells built on foundations with S=1.75 are, on average, less tilted compared to those with S=1.5 (under a one-tailed two-sample Welch's unequal variances t-test, t=-8.04, P<10-5). This trend suggests that cell size is not the only influential parameter and the tilt may be influenced by various structural factors of the hive, such as cell position on the frame, usage, content, etc."*

Line 190: "...a non-linear relationship..." --- I'm not sure "non-linear" is the best term here. Non-monotonic seems to be a better term.

We report the error from the linear fit model as described above in the updated version of Fig. 3 D and rephrase the non-linearity statement

Caption of Figure 3: "A) The 2D images of the frame with S = 1, 1.5, and 2. B) The 3D reconstruction of the sections of the comb, highlighted with a red box in panel A, using X-ray data, segmented to show comb in dark yellow

and the plastic in blue." --- Which is which? Indicate on the panels S = 1, S = 1.5, and S = 2.

We understand the confusion. We have reworked the figure and added the values of S to each panel.

Caption of Figure 3: "...frames with larger foundations" --- <frames with 1 <= S <= 2>.

We thank the reviewer for the suggestion, which has been implemented.

Lines 199-205: So, are you implying that the bees are using tilting as another compensation mechanism for suboptimal foundations (this time for S > 1)? If so, I suggest making this statement explicitly.

We have implemented the suggestion. In particular, we have described how naturally built drone cells (with no 3D printed foundation) are also more tilted than naturally built worker cells.

Line 212: "...they use the corners..." --- I suggest using <vertices> instead of corners (this is a better term, and also keeps it consistent with the discussion).

We thank the reviewer for the suggestion, which has been implemented.

Line 214: Again, use <vertices> instead of corners.

We thank the reviewer for the suggestion, which has been implemented.

Line 223: "...but quite similar to the cell sizes..." --- I suggest <but has a similar mean cell size>.

We thank the reviewer yet again for the careful reading of our manuscript. The suggestion has been implemented.

Lines 232-235: "It is worth noting that, while we printed the three-times-larger hexagon with a vertical orientation, with its side walls perpendicular to the top bar, the resulting honeycomb lattice built by bees features a horizontal orientation, with side walls parallel to the top bar." --- I found it difficult to understand this sentence. What is the "top bar"? What do you mean by "vertical" and "perpendicular" orientations? Please clarify.

We understand the confusion. We now identify one of the orientations as "*single-vertex-up*" (when

just one of the six vertices is pointing up, which is orientation most commonly observed in naturally built comb) and describe the other as a 30 degree rotation of this one.

> **Line 242: "...when S = 0.5, or S = 4" --- I saw no reference in the results to these sizes. Maybe worth mentioning briefly the outcome of S = 0.5 before elaborating on S = 0.75, and the outcome of S = 4 after elaborating on S = 3.**

We agree with the reviewer that the discussion was confusing. We have added a few sentences about S = 0.5 and 4, as the reviewer suggested. We added the following to the beginning of the Result section, before discussing S=0.75:

> *"We start our exploration by reducing the area of the 3D-printed cells on the foundations to half of the average worker cell size (i.e., S=0.5). Our findings suggest that when faced with such small foundations, bees do not follow the patterns or use the printed cells in any other form for comb building. The leftmost column in Fig. 1 D shows that bees fill most of the small printed cells with wax and build new hexagons on a mostly flat foundation. On the other end, increasing the size of the printed cells to four times greater than the size of the average worker cell (S=4) did not produce any consistent patterns (see the rightmost column in Fig. 1 D."*

We have also added the following paragraph to the results section:

> *"Within the range of cell sizes we examined, which is 0.5 ≤ S ≤ 4, it became apparent that bees do not effectively utilize the patterns on the provided foundation when S = 0.5, or S = 4. For S=0.5, the bees either disregard the printed pattern entirely or fill the small cells with wax (see Fig. 1 D). This allows them to initiate the comb construction on a flat foundation. This behavior resembles the nesting practices of wild honeybees found in tree cavities with numerous small cracks and crevices [37]. In such natural settings, bees tend to create a flat and waterproof surface by using tree resins (propolis) to seal and coat the tiny gaps and cracks, effectively preventing the growth of mold and bacteria [11]. In the case of S=4, the bees are unable to use the printed foundations to build a uniform structure. Most of the comb built on this size foundation seem to be randomly constructed in all directions, sometimes growing outwards perpendicular to the frame (see Fig. 1 D). We have also collected data using foundations with other sizes such as S=2.5 and S=3.5. On these frames we observed an inconsistent mix of tilted and layered hexagonal cells, see Fig.S6 on the Supplementary Materials section for examples."*

> **Lines 282-294: In journal paper discussions, I prefer statements like this phrased as "what can be done (by anyone)" and not as "what we will do", but this is somewhat a matter of taste.**

We understand the reviewers' point of view, but in this case, we will keep the original language, since we are describing on-going work in our project, instead of all possible research building on our results.

Response to Reviewer #3

> **The authors of this paper have used some recent technology to identify how bees respond to variation in surfaces when building their combs. While I think that there is some really interesting and valuable data presented here, the focus of the paper is, in my view, rather too focused on the technology than on the biological import of the data. In sum, the narrative here is dense and rather too distant from the biology to see how the authors have added substantially to what we know already. What would be useful (at least for this reader) would be for the authors to make their story more biologically accessible, otherwise there seem likely to be few who will see what they have added. It would help to be clearer about the problem and what is already known, and what questions this work specifically addressed.**
>
> **The authors currently describe the background as not having yet provided a thorough investigation into the extent of the adaptability of bees constructions and that they "shed light honeybees' adaptive comb construction strategies with potential to find applications in additive manufacturing, bio-inspired materials, and entomology." I am not really sure what this means: it currently seems too vague to be an especially valuable set of outcomes.**
>
> **The data seem sound enough but it is the biological interpretation that needs work and I think the authors need to make that clearer and more compelling before the substance of their work will be clear.**

We thank the reviewer for their positive comments, and for the encouragement to expand our discussion and make a stronger connection to biology.

We have tried to follow the suggestions and made extensive changes to the paper. These include adding references, rewording of discussion, as well as expanding the analysis of our results. In addition to the answers to other reviewers, our response includes a diff version, that highlights all our changes.

Response to Reviewer #4

The paper studies how honeybees 'adapt' their honeycomb building strategy to 'boundary conditions' in the environment. The authors use a clever 3D printing set-up as a 'seeding' of the honey comb pattern, and let the bees build upon it. Figure 1 summarizes the main result : for small and big pattern, the bees essentially neglect the prepattern, but for intermediate seeds, three behaviours are observed. For slightly smaller pattern than the 'natural' one, a 'merged' pattern is observed, where the prepattern is used as a template for merging cells to reach the normal size. For prepatterns slightly bigger (up to 2x) than the normal pattern, a 'tilted' pattern is used where, remarkably, the size distribution of the final cell sizes essentially follows the prepattern, but cells are constructed in a tilted way. When the prepattern is 3x as big as the 'normal' size, a clever 'layered' strategy is used, where, by and large, the printed pattern is used as a template for cells of normal size, centered at each of the 'corner' of the prepattern.

The study is very interesting and well-written, and I think it would be of interest for a general biology audience. I have the following comments/questions:

We thank the reviewer for their positive feedback and will respond to their questions below.

- For the merging case (Fig 2), is it possible to take the 3D x-ray, define cell size at a distance d from the prepattern, and to plot, say, the average cell size as a function of d? (basically along the coordinate X shown in 2F,H). I am wondering if the transition from 0.75 to 1 is continuous, or if there is a kind of distance threshold from which all cells get to size 1.

The reviewer presents a very interesting question. As shown in the Figure below, the transition is somewhat smooth, but with large differences from cell to cell. The same can be seen (although less clearly) in the video provided for the SI.

In short, in some cases the transition is very brief (barely over a millimeter) while for other cells it can be roughly five times longer.

We do not have sufficient data to answer this question right now in a conclusive manner. In particular, we need to cut the panels before we image them in the CT-scan (because otherwise they do not fit), so only small regions have been imaged. We are planning to perform more experiments, including other sizes in the $0.5 < S < 0.75$ range, and focus on this phenomenon.

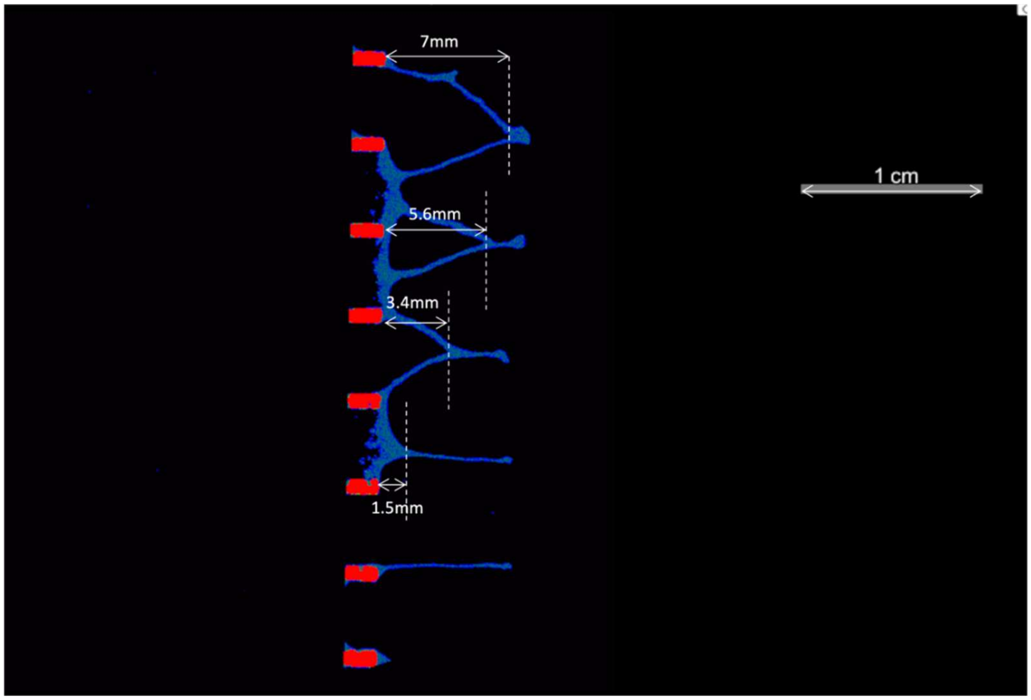

- For the same experiment, it is also interesting that most cells have in fact much bigger normalized size than 1. It is not clear to me why we see this : it suggests that the cells are first increased in size before being divided. Can the authors comment more on this ?

As discussed in our response to Reviewer 2, we do not have a good explanation for the differences in average. In particular, we do not know if they are limitations in our experiments (e.g., limited data or edge effects due to not having an infinite lattice) or a significant difference. We hope that future experiments will help us understand this mode of construction better.

- It seems to me that there are some 'magic numbers' (due to topology), where some special strategies might be used, and I am wondering if the authors could comment on this. For instance, it is striking to me that for a 1.5 size, the distribution of cell sizes is much narrower, and the tilt is much bigger than 1.25 and 1.75. In fact it seems closer to the natural drone comb.

It is indeed striking that there is not a monotonic trend in the average tilt of the cells. We believe that this can be due to the difference in the usages of cells. Cells built on the S = 1.5 are consistently used for drone comb. This can explain the narrow cell size distribution and why the tilt of the 1.5 cells are closer to the tilt of the natural drone cells. Whereas cells built on the S = 1.25 are sometimes used for raising workers and mostly for honey storage. This can explain why the tilt of the 1.25 cells are closer to the tilt of the cells with S=1, which is also primarily used for worker comb and honey storage. As for the S=1.75, even though the mean tilt is smaller than S = 1.5, the standard deviation is high (both for the cell sizes and for the tilts). This suggests a range of usages for these cells from drone comb (which require larger cells and higher tilts) to honey storage (which can be done in smaller cells that are less tilted), which is in agreement with our observations.

- Along the same line, for the layered strategy, 3 seems to be such a 'magic number', since the bees can exactly fit normal size hexagons at each corner. So by comparison, I think it would be good to discuss and quantify a bit more in the main text the cases where the geometry can not be easily accommodated. The 2.5 case should be especially interested, the authors mention there is a mix of titled and layeded hexagonal cells, but how 'mixed' is it? There could also be an interesting transition here to study, maybe for 2.8 the pattern is completely disordrered, and at 3 one gets something very regular

We again are limited by the amount of data available, since we only we only have a few panels available with this combination. We thank the reviewer for the interesting suggestion, and we will consider in experiments for Summer 2025, which we are currently planning.

- Along the same lines, I am wondering what happens if some 'disorder' is introduced in the prepattern. For instance, one could imagine more irregular hexagons, or with topological defects where from time to time 5-sided or 7-sided cells are introduced. The building strategy might changes in response to this (maybe defects are kept, or maybe transiently bigger cells are build to implement merging later). I have no good sense of how difficult it would be to do those experiments, but they could also be very informative.

We agree with the reviewer that the introduction of disorder or defective patterns could be interesting to explore. We tried 3D printing some panels with initial 5- and 7-sided cells randomly scattered on the panel, but the printing process was unsuccessful this year, so we did not use them in our experiments. We have some ideas on how to fix them, but since the experimental bee season has passed, the earliest we can try running these experiments will be Summer 2025.

Adaptive Cell Size, Merging, Tilting, and Layering in Honeybee Comb Construction

Golnar Gharooni-Fard[1], Chethan Kavaraganahalli Prasanna[1],
Orit Peleg[1,3-7*], Francisco López Jiménez[2*]

[1]Department of Computer Science, University of Colorado Boulder, CO, USA,
[2]Department of Aerospace Engineering, University of Colorado, Boulder, CO, USA
[3]Department of Physics, University of Colorado Boulder, CO, USA
[4]Department of Applied Math, University of Colorado Boulder, CO, USA
[5]Department of Ecology and Evolutionary Biology, University of Colorado Boulder, CO, USA
[6]BioFrontiers Institute, University of Colorado Boulder, CO, USA
[7]Santa Fe Institute, NM, USA

[*] Orit.Peleg@colorado.edu [*] Francisco.LopezJimenez@colorado.edu

Short title: Bee Honeycomb Construction for Different Cell Sizes

Honeybees are renowned for their skills in building intricate and adaptive hives that display notable variation in cell size. However, the extent of their adaptability in constructing honeycombs with varied cell sizes has not been thoroughly investigated. We use 3D-printing and X-ray ~~Microscopy~~ microscopy to quantify honeybees' capacity in adjusting the comb to different initial conditions. Using the average area of natural worker cells as a reference, our findings suggest three distinct construction modes when faced with foundations of varying cell sizes. For smaller ~~cell sizes~~foundations, bees occasionally merge adjacent cells to compensate for the reduced space. However, for larger cell sizes, the hive uses adaptive strategies like tilting for foundations with cells up to twice the reference size ~~,~~ and layering for cells that are three times larger than the reference cell. Our findings shed light on honeybees adaptive comb construction ~~strategies with potential to find applications in additive manufacturing,~~ modes, significant for the biology of self-organized collective behavior, as well as for bio-inspired ~~materials, and entomology~~engineered systems.

Introduction

Honeybee nests are the emergent outcome of the collaborative efforts of thousands of individual bees. These structures play a vital role in the survival and functioning of a bee colony by providing storage space for nectar and pollen, a nursery for brood development, and a structurally stable environment for the various interactions among the nestmates (*1, 2*). The hexagonal ~~structure of the honeycomb~~ shape of honeycomb cells, built with a near-optimal minimization of the wax-to-storage space ratio (*3*–*5*), has fascinated scientists for millennia (*6*–*8*). This optimization is necessary because wax production incurs a high

energy cost (*9*). In particular, to secrete just 1 *lb* (454 *g*) of wax, honeybees need to consume about 8 *lbs* (3.8 *kg*) of honey (*10*).

~~However, it has been shown that feral bees~~ Feral bees have been shown to construct their nests on a range of surfaces such as tree branches and pre-existing ~~tree~~ cavities, which do not necessarily allow for ~~perfect~~ regular hexagonal lattices (*11*). ~~Additionally,~~ Furthermore, comb building is a distributed process with no global blueprint for bees to follow. Instead, workers often initiate ~~the process of comb building~~ constructing the comb simultaneously from multiple locations within the hive~~simultaneously (*12*)~~ (*13*). To achieve a coherent structure, ~~the~~ separate pieces of comb need to be merged ~~,~~ and workers must reconcile the differing geometries of cell sizes at the boundaries (notably in terms of size and alignment), while conforming to the available space and resources (*14*). The resulting structure inevitably contains non-regular hexagons and topological defects with various cell sizes, to adapt to environmental constraints (*13, 15–17*). ~~Adjusting the sizes~~

Adjusting the shape, size, and arrangement of hexagons during honeycomb construction may serve ~~additional~~ multiple purposes, often dictated by the colony's requirements. ~~The need for these adaptations may arise, for instance, when the colony requires space to nurture workers or drones, or to store honey, among other reasons (*13*). Despite the frequent occurrence of these size modifications,~~ Previous studies across various honeybee species have shown the precise design of their nest, optimized to maximize storage capacity and brood space (*10, 18, 19*). These adaptations occur as the needs of the colony fluctuate, requiring more or less space for the rearing of the ~~degree of adaptability and variability in the structure of the honeycomb in 3D has not been thoroughly investigated.~~ brood or the storage of honey over time (*13*). It is worth noting that honeybees are not the only species that adjust their nest structure based on external changes in the environment and colony requirements. Social wasps have also been shown to use architectural solutions to build their nests, similar to honeybees (*20, 21*). The combs of paper wasps exhibit remarkable adaptability, with structural variations influenced by factors such as nest maturation, overall size, and cell arrangement (*20, 22, 23*).

More than four decades ago, H. R. Hepburn (*10*) studied the comb con-

struction by African Honeybees *Apis melifera adansonii*, providing them with sheets of beeswax foundations having a range of cell sizes from 170 to 1022 $cells/dm^2$ (between 1 to 6 times the average size of ~~cells built by bees with no constrains~~the natural honeycomb cell). Using 2D images of the built comb in the experimental frames, the study reported various construction solutions and measured ~~working tolerances from the comb built on the foundations~~ the working tolerances of the comb with variable cell sizes. Yet, honeycomb represents a three-dimensional structure, and the details and intricacies of it cannot be fully discerned and quantified using two-dimensional images. While the specific behavioral rules of individual bees during comb building remain unclear, revealing the 3D structure of the comb built under various initial conditions will be a crucial step in linking the local behavior to the global building patterns. For instance, the 3D progression of the nest building process, shown in a recent study by Marting et al (*12*), reveals that bees prioritize building a well-connected nest of parallel combs that remains structurally and functionally robust, even when faced with experimental disruptions. Such findings show the importance of accounting for 3D comb structure and provide valuable insights into the adaptive and resilient nature of the honeybee superorganism.

In this work, we study the adaptability of the comb construction by European honeybees *Apis melifera* L, under different initial conditions, using 3D visualization. We leverage 3D-printing to design repeatable experiments with precisely controlled and carefully quantified cell areas as ~~starter sheets.~~ foundations. By exploring the responses of honeybees to various initial conditions (using foundations with different cell sizes), our objective is to exhibit the range of construction modes bees use to build their nest, as well as the complexity of these structures and their sensitivity to initial conditions. Our findings corroborate and expand upon prior research by delineating the distinct construction modes employed by bees in response to ~~various initial conditions regarding~~ specific cell sizes. ~~Furthermore, to~~ To highlight the details of each construction mode, we use X-ray microscopy (XRM) to quantify the properties and visualize these intricate structures. ~~Recently~~In a recent study, Franklin et al (*24*) have also used XRM technology to describe a step-by-step process by which honeycomb is constructed in natural settings which sheds light on the ~~intricate structure of~~

~~honeycomb cells as they are being builtin the absence of a foundation. However, our focus is on identifying various methods of constructing combpieces, reflecting bees'ability to adapt to different enforced cell sizes through intricate three-dimensional structures. To that end, our method pairs XRM with 3D-printing, to bring out these various construction modes and their complexity and adaptability to specific initial conditions with variable cell sizes~~structure of a natural honeycomb cell as it is being built. Their findings contribute to understanding the nest construction mechanism by showing how individual cells grow directly from the hexagonal pattern on the comb's spine, enhancing structural stability through staggered lattice patterns and strategic material deposition. Our work examines how different cell properties and arrangement give rise to distinct patterns and how these patterns can be interpreted in relation to the initial conditions on which they are built.

To distinguish various foundations, we define the cell size variable (S) as a multiple of the inner area of the average honeybee worker cell, ~~which is $12.5\ mm^2$. This value is~~ that is derived from the length of a regular hexagon, which we measured to be ~~$4.4\ mm$~~ 5.4 mm on a free comb built ~~with no~~ without a foundation. Fig. 1A shows a schematic of the shapes of the cells built on the plastic base with $S = 1$, equal to the size of an average worker cell. Fig. 1B shows two snapshots of a 3D-printed frame with $S = 1$ at the start and end of the comb building process. Fig. 1C shows the 3D-visualization of the structure of the comb using X-ray data from a section of the frame shown in panel B. The details of how we process the raw X-ray data to generate 3D-visualizations of the comb structure are described in the Methods Section. Using ~~this size as our baseline~~$S = 1$ as our control, we supply the hives with custom 3D-printed frames with regular hexagonal patterns of various cell sizes, ranging from half to four times the size of a reference cell (*i.e.*, $0.5 \leq S \leq 4$). Our goal ~~with~~ in changing the foundation's cell sizes is to examine the bees' capacity to adjust the comb structure to ~~consistently smaller or larger cells~~the given foundation. Fig. 1D provides an overview of our results, highlighting different construction modes observed on our 3D-printed frames. It includes photographs of ~~these building modes,~~ the obtained structures followed by schematics ~~for~~ of adaptive strategies used for honeycomb construction with varying cell sizes. ~~The details~~

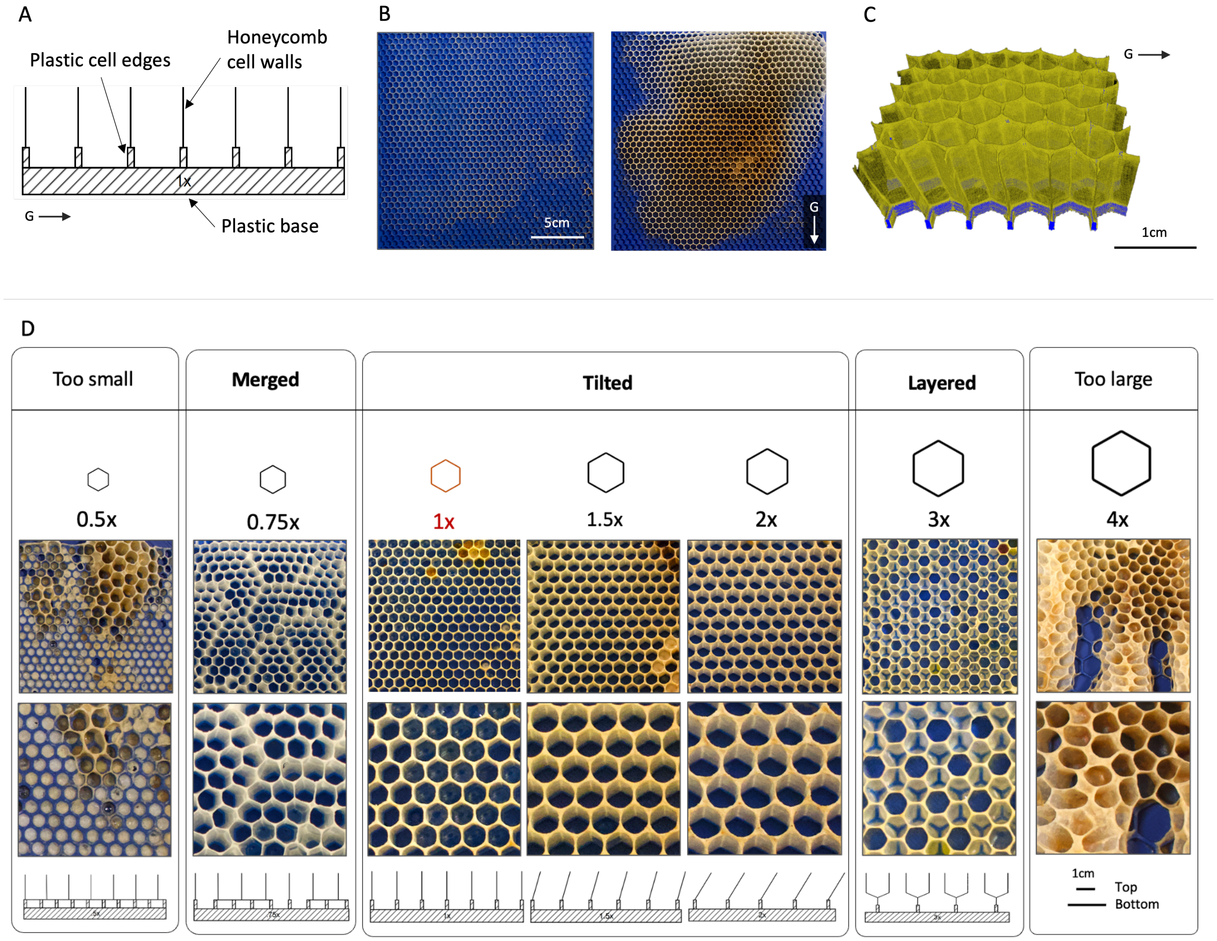

Figure 1: A) Schematic of the honeycomb cells built on a control frame with $S = 1$. B) Two snapshots of the initial frame with the plastic, 3D-printed foundation along with the same frame after 20 days. The printed cell size on this frame is $S = 1$. C) XRM-based 3D-visualization of a section of the frame with $S = 1$, segmented to highlight the comb in dark yellow and the plastic in blue. The direction of gravity is indicated by the arrow ~~labelled~~ labeled G. D) Overview of the different modes of honeycomb construction on 3D-printed frames with varying cell sizes. The top row shows the building mode in relation to the cell sizes on each foundation, displayed beneath them. The subsequent row presents images of the constructed honeycomb on the foundations, accompanied by a zoomed-in view beneath. The bottom row illustrates schematics depicting adaptive strategies for honeycomb construction with differing cell sizes.

~~of the structure of the honeycomb in each mode is discussed in the following section.~~

Results

We begin this section by discussing the different construction modes identified in our experimental data, followed by a more detailed description and visualization of the overall structure of the comb in each mode. We ~~specifically~~ start our exploration by reducing the area of the 3D-printed cells on the foundations to half of the average worker cell size (*i.e.* $S = 0.5$). Our findings suggest that when faced with such small foundations, bees do not follow the patterns or use the printed cells in any other form for comb building. The leftmost column in Fig. 1 D shows that bees fill most of the small printed cells with wax and build new hexagons on a mostly flat foundation. On the other end, increasing the size of the printed cells to four times greater than the size of the average worker cell ($S = 4$) did not produce any consistent patterns (see the rightmost column in Fig. 1 D. However, within the range of $0.75 \leq S \leq 3$, we identify three different building modes—merging, tilting, and layering—relative to the ~~given foundationsizes~~size of the printed foundation. The intricate properties of each construction mode, along with 3D-visualization using XRM, ~~is~~ are presented in the following sections.

Merging

~~To examine how bees adapt their comb structure when faced with smaller foundational cell sizes , we manipulate the cell areas of the printed patterns, reducing the area of the 3D-printed cells to 75% the~~ When faced with foundations where cell sizes are 0.75 times smaller than the size of an average worker cell (~~*i.e.*~~ $S = 0.75$), bees adapt the comb structure and use the foundation with an unexpectedly intricate approach. Fig. 2 ~~A-D illustrates the evolution of the~~ A–D illustrates the progression of comb construction on one ~~of these experimental frames~~ such experimental frame over a 20-day period. There is an apparent pattern at work from the early stages of comb construction, indicating the swarms' ~~consistent~~ recognition of and adaptation to this new cell size pattern. As shown in the insets of Fig. 2~~A-B~~ A–B, even though most of the ~~initially~~ drawn hexagons start from the plastic edges on the printed foundation, some cell walls are built with an angle that ~~will eventually merge~~ eventually merges with the walls ~~from the~~ of

adjacent cells built with a reverse tilt. This pattern repeats ~~occasionally until the whole~~ until the entire frame is covered with hexagonal cells. The term "merging" describes the occasional combination of several small cells to create the necessary space to construct natural worker-size hexagons.

Fig. 2~~D~~ C shows the frame after ~~the~~ comb construction is complete, but prior to ~~the~~ filling of cells with honey or brood, at which point X-ray imaging is conducted. We find that the merging behavior during honeycomb construction is not specific to our experimental frames with smaller printed foundations. A similar pattern can sometimes be observed on the comb built on some of the commercially available frames with worker-size foundations (equivalent to our 3D-printed frames with $S = 1$). Fig. 2 D shows an image of the comb built on one of the commercial frames inside our hives using the merging mode. This adjustment may be made to meet the colony's specific needs for constructing larger cells, such as those used for raising drones. The series of images in Fig. 2~~E-H~~ E–H demonstrate the X-ray results. In particular, Fig. 2 E and Supplementary Movie S1 illustrates a 3D-reconstruction of a 5 ~~cm~~ cm $\times$ 5 ~~cm~~ cm section of the frame in Fig. 2~~D~~ C, highlighting plastic in blue and comb in dark yellow. To better visualize the merging process, the cross-sections of the X-ray are presented in Fig. 2F-H, using planes containing two of the Cartesian axes, shown in green (XZ), blue (YZ), and red (XY) in Fig. 2 E. For ~~instance, the positioning of the cell walls on the XZ slice shown~~ example, the XZ slice in Fig. 2~~F highlights how~~ F shows how the comb material is used to ~~combine~~ join adjacent plastic edges beneath the cells~~constructed on top of them~~.

~~Additionally, in the bottom view of the sample in Fig. 2I, it can be observed that some of the printed cells are entirely covered with comb. This coverage aims to provide additional space for the initiation of a normally-sized hexagonal lattice. The distribution of covered cells on a larger section of the frame can be viewed in Fig. 2J, with covered cells shown in gray. Interestingly, we find that on average, one in four cells built on the frames with $S = 0.75$ foundation is covered. This ratio suggests that bees employ the "merging" strategy to effectively transform a slightly small hexagonal grid into their commonly-used size. In other words, they combine one out of four cells to compensate for the 25% less space imposed by our 3D-printed foundations with $S = 0.75$.~~

Remarkably, while some of the printed cells are filled with wax, the top view of the comb structure, shown in Fig. 2 G, still features a regular hexagonal grid with cell sizes that are comparable to the average worker cell size. ~~For comparison,~~ Fig. 2 K depicts the distribution of ~~cell sizes~~ normalized cell size for cells built on ~~the~~ frames with $S = 0.75$ in blue, ~~along with the distribution of the reference cell sizes shown in gray. The blue distribution covers a similar region but~~ and the same for reference cells ($S = 1$) in orange. The normalized cell size is computed by dividing the built cell areas by the mean reference cell area. For better interpretability, we will use normalized cell sizes in future discussions, unless otherwise stated. As depicted in Fig. 2 K, the mean normalized cell size built on foundations with $S = 0.75$ is 0.09 units larger than the mean reference cell size, but 0.34 units larger than the area of the corresponding printed cells $S = 0.75$. This suggests that on foundations with $S = 0.75$, bees are building honeycomb similar to the typical cell size that is closer to the reference cells than to those on the printed foundation. Moreover, apart from being slightly larger than the reference cell size ($\mu_{\mathrm{S=0.75}} = 1.09, \mu_{S=1} = 1, t = 44.0385, \mathrm{P} < 10$[illegible] for a one-tailed two-sample Welch's unequal variances t-test), the variation in cell size for these cells is considerably wider (~~$S = 1.04 \pm 0.02$ versus $S = 1.12 \pm 0.11$~~ $\sigma_{\mathrm{S=0.75}} =$ [illegible] $\sigma_{S=1} = 0.03, W = 971.173, \mathrm{P} < 10^{-5}$ for the Levene test to compare variances), suggesting less consistency in the cell sizes of the comb built on the ~~foundation with $S = 0.75$~~ smaller foundations. The higher standard deviation could be attributed to the discrepancy between the ~~given~~ printed and desired cell sizes and the inherent randomness of the merging process. ~~It is worth noting that the merging behavior during honeycomb construction is not specific to these experimental frames with smaller given foundations. A similar pattern can sometimes be observed on the comb built on some of the commercially available frames with worker-size foundations (equal size to~~ See Table S1 in the Supplementary Materials section for more details on the number of samples in each category, the individual sample size and statistics.

Additionally, in the bottom view of the sample in Fig. 2 I, it can be observed that some of the printed cells are entirely covered with comb. This coverage potentially aims to provide additional space for the initiation of the hexagonal lattice, with $S \approx 1$, which, as noted earlier, is what bees construct on the $S = 0.75$

foundations by merging cells. The distribution of covered printed cells on a larger section of the frame can be viewed in Fig. 2 J, with covered cells shown in gray. If we hypothesize that bees compensate for the 25% reduction in space per cell (relative to their average worker cell size) by combining cells in our 3D-printed frames with $S = 1$) . See Fig. S3 foundations with $S = 0.75$, we would expect them to cover 25% ($h_0 = 0.25$) of the cells in the printed foundation to build a hexagonal lattice with $S \approx 1$. Interestingly, all individual experimental samples, as well as the pooled data, fail to reject our null hypothesis ($h_0 = 0.25$) under a two-tailed one-sample z-test for proportions at a significance level $\alpha = 0.05$ (when pooled together: $h_{\text{pooled}} = 0.24$, z $= -0.744$, P $= 0.457$). For details about the individual replicates, see Table S2 in the Supplementary Materials for an image of the comb built on one of the commercial frames inside our hives using the merging strategy . This adjustment may be made to meet the colony's specific needs for constructing larger cells, such as those used for raising dronessection. This suggests that bees employ the merging strategy to effectively transform a slightly smaller hexagonal grid ($S = 0.75$) into their commonly used size ($S = 1$), and the behavior has been consistent across our samples.

Tilting

The next mode of construction corresponds to the case with the given printed foundation of cells with $1 \leq S \leq 2$, where bees use the provided patterns but increase the tilt angle of the cells as a function of built cells as the cell size increases. Fig. 3 A presents a series of 2D-images 2D images of the comb built on frames with $S = 1, 1.5$, and 2. The corresponding 3D-reconstructions 3D reconstructions of the comb built on these frames are presented in Fig. 3 B, which are the result of scanning sections of the combs highlighted with red boxes in Fig. 3 A. Tracing the plastic edges shown in blue and the comb in dark yellow across the three samples displayed in Fig. 3 B reveals a consistent behavior: bees construct honeycomb cells along the edges of plastic follow the printed foundations, regardless of the increasing cell sizesobserved in the samples. .

The distribution of the built cell sizes size of the cells built on frames with

foundations ~~ranging from 1 to 2, as depicted~~ with $S = 1, 1.5, 2$ is shown in Fig. 3~~C, demonstrates a monotonic increasing trend indicating a clear positive correlation~~ C, with their detailed statistics shown in Table. 1. A linear least-squares regression line that fits the cell size data, using normalized cell size as the response and the foundation size as the predictor, has a positive slope that is statistically significant ($\beta = 0.89, P < 10^{-5}$), demonstrating an increasing trend of built cell sizes with the foundation size. Moreover, Fig. 3 C suggests that this increasing trend in cell sizes is monotonic, with the strength of the trend highlighted by a high correlation coefficient (Pearson's correlation coefficient $\rho = 0.97, P < 10^{-5}$), indicating a strong positive relationship between the foundation and the final cell size. This ~~confirms~~ provides strong evidence of the bees' ability to effectively utilize the ~~provided patterns~~ printed foundations by adjusting honeycomb cell ~~sizes to match the given foundations~~shapes and sizes in response to structural changes. According to our observations (which are consistent with previous findings (*14, 25–27*)), these larger cells ~~that are too large~~ (built on foundations with $1 < S \leq 2$) are not suitable for raising workers, ~~are~~ which are normally raised in cells built with $S \approx 1$. Therefore, these larger cells are either used for raising drones or storing honey~~(see~~. See Fig. S4 for some examples ~~)~~of the usages of these cells in our experimental frames. For context, the ~~dashed line in Fig.3C marks the average cell size of natural drone brood cells. While the dashed line aligns well with the $S = 1.75$ and closely follows the $S = 1.5$ distributions, it is quite far from $S = 2$. Nevertheless, it is interesting to observe that in our experiments all of the larger cell types are utilized for drone rearing when required~~average drone cell size (*28*) when normalized is equal to $S = 1.61$.

~~Additionally, we notice that the bees notably~~ As shown in Fig. 3D, we find that bees significantly increase the tilt of cells as the foundation ~~'s cell~~ sizes increase. Previous studies exploring the natural tilt of ~~the~~ honeycomb cells have indicated that a standard worker cell tilts up to 13° (*15, 28, 29*). It has also been established that the direction of this tilt is influenced by gravity~~, meaning that~~: bees consistently tilt ~~the~~ cells upward (*18, 30, 31*)~~, against the direction of gravity~~. In our experiments, we note that cells on the control frames with $S = 1$ are tilted, on average, by ~~8.75°~~ 8.74° against the direction of gravity.

For comparison, we computed the average tilt angle of natural drone cells to be 20° (see Fig S5 for more information). The tilt of the drone comb is measured manually using images of natural drone comb built on a flat surface, such as Fig. S5. The mean angle of tilts, their 95% confidence intervals and standard deviations for each foundation size $1 \leq S \leq 2$ are shown in Table 1shows the average tilt .

Fig. 3D shows the tilt distribution of the comb cells built on various foundation sizes, along with a best-fit linear regression line. It is evident that there is a gradual rise increase in cell tilts with increasing values of S. However, with a linear least-squares regression line fit to the angle data (response: angle of tilt, predictor: foundation size) showing a statistically significant positive slope ($\beta = 17.36, P < 10^{-5}$). Although the linear positive trend is strong (Pearson's correlation coefficient: $\rho = 0.84, P < 10^{-5}$), this relationship is not strictly linear. Notablymonotonic. In particular, cells built on foundations with $S = 1.75$ are, on average, smaller than less tilted compared to those with $S = 1.5$ and they also exhibit a wider range of tilts (with a standard deviation of 2.73°(under a one-tailed two-sample Welch's unequal variances t-test, $t = -8.04, P < 10^{-5}$). This trend suggests a non-linear relationship between cell size and tilt, indicating that tilt that cell size is not the only influential parameter and the tilt may be influenced by various hive structural factors structural factors of the hive, such as cell position on the frame, usage, content, etc. For comparison, the last row in Table 1 shows the tilt angle distribution for natural drone cells. or content.

Prior Previous studies have suggested various hypotheses to explain the natural tilt of the honeycomb cells. Mullenhoff (*32*) posited that this tilt prevents honey outflow. However, a recent study by Oeder et al. (*29*) challenges this idea, proposing instead that the tilt redistributes weight onto the midwall, potentially increasing the comb's carrying capacity. In Additionally, in our experiments, we find that the amount of tilt effectively reduces the cross-sectional area of the cell, when considered perpendicular to the walls. For example, when considering the case of When we cut the cells of the sample built on foundations with $S = 2$ and cutting the sample with using a plane perpendicular to the walls at the top of the sample, we find that the cross-sectional area of the hexagons at in that layer is, on average, 0.77 times smaller than the area of the printed hexagons

. This means that at its bottom. In other words, the cross-sectional area is equivalent to $S = 1.54$, a significant reduction with respect to the provided lattice compared to the size of the provided foundation $S = 2$. Moreover, these tilted cells also have a larger depth, defined along the direction of the walls, for a given total volume of the cell. In general, the angle of tilt could represent a compromise between maximizing volume, ensuring structural stability, and optimizing construction efficiency. See Supplementary Movies S2-6 for the 3D reconstruction of all the samples built with tilting cells.

heightFoundation cell size (S) **Foundation cell size (S)**	**Average normalized cell size** **Normalized cell size**		[illegible]
	**Mean** (μ)	**Std. dev.** (σ)	95
height1	1.04 +/- 1	0.03	[1.00
1.25	1.24 +/- 1.19	0.09	9.44 +/- 1.9
1.5	1.55 +/- 1.49	0.06	[1.48
1.75	1.67 +/- 0.11 1.6	16.7 +/- 2.73 0.1	[1.60
2	1.97 +/- 0.08 1.89	0.07	[1.89

Table 1: Mean and standard deviation of cell sizes and tilts measured across various given printed cell sizes (S).The tilt for the drone comb is measured manually using images of natural drone comb built on a flat surface, such as Fig. S5.

Layering

In this section we explore the Lastly, we explore cells built on frames with cells having areas three times larger than those of worker cells (*i.e.* $S = 3$), which result in leads to a new mode of construction, ; see Fig. 4. Contrary to the previous cases, the bees did Unlike the previous case, bees do not adapt the provided pattern by simply constructing larger or simply by constructing larger and more tilted cells. Instead, they use the vertices of the 3D-printed cells as the foundation for building a new layer of cells, forming a two-layer structure; see Fig. 4 A. This 2D view shows that the cells on the upper layer are centered at the vertices ability to build cells that are centered and supported by the edges of the previously built cells reflects their natural behavior. Fig. 4 B shows a piece of a two-sided comb from a hive without foundation, where the front side is built upon the structural support of the pre-existing lattice on the back. The

geometry of the 3D-printed ~~hexagons. This arrangement results in~~ panels in our experiments resulted in the emergence of an additional cell, located at the center of the ~~provided hexagons, though smaller in size~~printed hexagons. This cell extends ~~until~~ to the base of the 3D-printed frame, merging with the ~~provided hexagon. This structure becomes more clear~~ printed hexagon. The positioning and structure of the cells are more clearly illustrated in the 3D view of the comb ~~, depicted~~ shown in Fig. 4~~C and Supplementary Movie S7 that shows~~ C, highlighting how the honeycomb cells (~~shown in~~ dark yellow) are ~~positioned on top of~~ built atop the plastic edges (~~shown in~~ blue).

~~As shown in~~ Fig. 4~~B,~~ D depicts the distribution of ~~final cell sizes is wider (the standard deviation is 0.02 for~~ normalized cell sizes of cells built on foundations with $S = 3$ and the reference cells ($S = 1$~~vs. 0.07 for $S = 3$), but has a similar mean cell sizes compared to~~), with their means and standard deviations included. We find that the mean normalized cell size of cells built on foundations with $S = 3$ is 0.06 units smaller than the mean of the normalized reference cells, whereas these cells are 2.06 units smaller than the printed cells in the $S = 3$ frames, showing that cells built on foundations with $S = 3$ are much closer in size to reference worker cells than to their printed foundations. Typically, these cells are smaller than reference worker cells ($\mu_{S=3} = 0.94, \mu_{S=1} = 1, t = -26.67$, P $< 10^{-5}$ for a one-tailed two-sample Welch's unequal variances t-test), while the variation in their normalized cell sizes is wider than that of the reference cell size distribution ($\sigma_{S=3} = 0.06, \sigma_{S=1} = 0.03, W = 285.59$, P $< 10^{-5}$ for the Levene test to compare variances). The similarity in size of the cells built on ~~the control frames. This~~ $S = 3$ foundations to that of the reference cells suggests that the two-layer strategy employed by bees effectively transforms the ~~given~~ printed foundation of $S = 3$, which is ~~too large to serve~~ not suitable for any purpose in the hive, to $S = 1$, which is the most commonly used.

Overall, this symmetrical structure ~~is comprised of six new normal-size hexagons superimposed on the three-times larger given hexagon, resulting in the formation of another hexagon at the~~ shown in in Fig. 4 A, is composed of six newly constructed hexagons layered over the original foundation, forming another hexagon in its center. Consequently, as ~~shown in~~ illustrated in the zoomed-in image in Fig. 4 A, each gray hexagon ~~on~~ in the top layer derives ~~about 1/3~~

approximately one-third of its area from the 3D-printed (blue) cell and about 2/3 the remaining two-thirds from the neighboring cells upon on which it is constructed. It is worth noting that, while built. Another notable aspect of this structure is the change in lattice orientation during this geometric transformation. Although we printed the three-times-larger hexagon with a vertical orientation, with its side walls perpendicular to the top bar, the resulting honeycomb lattice built by bees features a horizontal orientation, with side walls parallel to the top bar.hexagons on the foundation with a single-vertex-up orientation, the honeycomb lattice constructed by the bees exhibits a 30° rotated structure (as shown by the blue and gray hexagons in Fig. 4 A). While the former orientation is commonly used in commercially-available commercially available foundations and it is the orientation most commonly observed in naturally built comb, the latter has been found to be approximately 30% stronger structurally (*10*). Further increase in the printed cell sizes (*e.g.* $S = 4$) did not produce any new patterns. We will discuss more details about all the tested patterns in the following section.

Discussion

The flexibility of comb building behavior in bees has been previously discussed as a result of stigmergy (*33*) combined with a highly rich behavioral repertoire owned by bees (*34*). Our experimental results extend our understanding of honeybees' remarkable comb-building adaptability, providing detailed evidence of their diverse construction techniques when faced with varying foundation cell sizes. Our findings align with and strengthen previous research by introducing more variety of construction modes in response to specific environmental conditions. The observed variety of solutions might suggest cognitive planning (*35, 36*) –where bees potentially maintain a mental template of the desired outcome and select appropriate building modes based on foundation geometry–our study primarily focuses on how bees demonstrate multiple effective strategies for managing geometric challenges related to cell size, while maintaining structural functionality. However, the cognitive aspects of forward planning at the colony level would require dedicated experiments that are beyond the scope of this

paper.

Within the range of cell sizes we examined, which is $0.5 \leq S \leq 4$, it became apparent that bees do not effectively utilize the patterns on the provided foundation when $S = 0.5$, or $S = 4$. For $S = 0.5$, the bees either disregard the ~~provided~~ printed pattern entirely or fill the small cells with wax (see Fig. 1D). This allows them to initiate the comb construction on a flat foundation. This behavior resembles the nesting practices of wild honeybees found in tree cavities with numerous small cracks and crevices (*37*). In such natural settings, bees tend to create a flat and waterproof surface by using tree resins (propolis) to seal and coat the tiny gaps and cracks, effectively preventing the growth of mold and bacteria (*11*). In the case of $S = 4$, the bees are unable to use the ~~given~~ printed foundations to build a uniform structure. Most of the comb built on this size foundation seem to be randomly constructed in all directions, sometimes growing outwards perpendicular to the frame (see Fig. 1D). We have also collected data using foundations with other sizes such as $S = 2.5$ and $S = 3.5$. On these frames we observed an inconsistent mix of tilted and layered hexagonal cells, ~~please~~ see Fig. S6 on the Supplementary Materials section for examples.

For the remaining foundations tested ($0.75 \leq S \leq 3$) our results show different mechanisms by which honeybees adapt regular hexagonal foundations of different sizes to ~~satisfy~~ respond to the imposed structural changes while satisfying their needs. In the cases with $S = 0.75$ and $S = 3$, the provided cell sizes are ~~too~~ quite different from those naturally used by ~~the~~ bees, so they need to adapt them to build a new lattice. In the case of smaller cells, the size mismatch between both layers is resolved by effectively blocking some of the ~~given~~ 3D-printed cells, so that their space is distributed among their neighbor cells. This can be understood as the discrete version of systems in which geometric mismatch between two layers is resolved through localization of deformation, from cracks in drying films or mud (*38, 39*) to ridges in pre-stretched elastic bilayers (*40,41*), which result in similar patterns to those created by the blocked hexagonal cells. In the case of $S = 3$, the bees use the plastic edges of the provided frame as a foundation to build a new regular hexagonal lattice. The new hexagons are centered on the edges and vertices of the provided lattice, constructing a new layer of hexagonal lattice with cell size close to $S = 1$. This

structure closely resembles the comb construction seen in African honeybees on a foundation documented in (*10*). While Hepburn described this middle cell as a "false" cell that is not easily noticed in the completed comb, our experiments reveal that the broad base of this central cell can serve as an additional storage space for honey and pollen. In contrast, in the range $1 \leq S \leq 2$, the bees extend the provided lattice, constructing larger cells while increasing the tilt of the cells as their size increases. For a comprehensive comparison of the cell sizes constructed on all provided foundations, please refer to Fig. S7 in the supplementary materials section.

In our previous work (*17*), we introduced a 2D model capable of replicating the patterns and defects ~~on~~ in the comb structure arising from various geometric frustrations. With the identification of novel building modes, particularly the two-layered pattern in our current study, ~~now~~ we are motivated to extend our 2D model into a 3D framework. This extension will enable us to delve deeper into understanding the potential costs and benefits associated with such structural adaptations, which could be leveraged in the design of novel lightweight structures conforming to complex irregular geometries. Furthermore, given the bees' demonstrated capacity to construct and utilize large tilted cells, our future work involves rationalizing the relationship between cell size and angle, starting with the two reasons already proposed in the literature. With that objective in mind, we will examine the mechanical stability of the comb under larger tilt values, as well as the fluid mechanics that govern honey outflow within these structures. Future work could also test the response of the bees to foundations with lattices introducing some added disorder or defective patterns such as non-hexagonal cells. These future directions aim to enhance our comprehension of the intricate dynamics underlying comb construction and functionality of these adaptive structures.

Methods

To collect data about the structure of the comb, we establish a set of behavioral assays combined with X-ray imaging. The focus of this study is on the cell size $(0.5 \leq S \leq 4)$ which is varied systematically using 3D-printed panels so that

its effect can be isolated and quantified. The design work for preparing the 3D-printed frames is conducted using the SolidWorks 2019 CAD design software. All of our experiments are performed using colonies of European honeybees *Apis melifera* L. at the Peleg lab apiary in Boulder, Colorado. We use langstroth hives with 19~~*in*~~ in (480~~*mm*~~ mm) frames, with $9\frac{1}{8}$~~*in*~~ in (230~~*mm*~~ mm) depth, and $1\frac{3}{8}$~~*in*~~ in (35~~*mm*~~ mm) width. Each frame consists of a pair of 3D-printed plates of the size 212×212~~*mm*~~ mm that are placed opposite to each other. For our control set with $S = 1$, each hexagonal element on the 3D-printed plates has a side-length of 2.7~~*mm*~~ mm and outer area of 18.94~~mm^2~~ mm^2 (including the cell walls). All the other configurations are designed relative to the area of our control set. To confirm the observed modes of construction, we collected at least three repetitions of samples for each cell size, S. See the Supplementary Data section of the Supplementary Materials for pictures of these samples. To account for at least three repetitions of each of the configurations under study, we scatter a total of ~~50~~ 50 3D-printed experimental frames into ten honeybee colonies and fill the rest of the space in the hives with normal frames with full plastic foundations. In general, each colony has half 3D-printed frames and half normal frames.

To stimulate comb building, we apply a fine layer of natural beeswax to the printed foundation. The comb construction progress is monitored through regular photography sessions, capturing images of both sides of the experimental frames. To ensure minimal disruption during photography, we set up our photography station within the apiary, allowing us to quickly return the frames to their original locations inside the hives. Once the experimental frames are fully built, they are carefully extracted from the hives, and kept in the lab for further analysis. We obtain high-quality images of the comb using a Nikon DSLR camera and controlled lighting, and a black background. The camera is mounted on a fixed metal structure 2~~*m*~~ m from the comb to avoid distortion at the edges of the image and to keep a constant pixel-to-millimeter relationship. At this distance and camera resolution, 1~~*mm*~~ mm = 15 pixels.

To further analyze the honeycomb structures obtained after the completion of the experiments, we used 3D X-ray microscopy (XRM) to scan 5~~*cm*~~ cm $\times$ 5~~*cm*~~ cm sections of the comb constructed on our experimental frames. This pro-

vides a series of high resolution cross-sections of pieces of the comb that can be combined to generate a 3D virtual model. All tomography scans are performed using $\mu - CT$ ZEISS Xradia 520 Versa (Carl Zeiss X-ray Microscopy Inc, Pleasanton, CA, USA). The X-ray source is set to voltages in range 80-90~~*kV*~~ kV and power 7~~*W*~~ W, while the detector camera is set with an exposure time of 2 seconds for each of the 2034 images captured by optical magnification 0.4. Image processing and segmentation on the X-ray data are conducted using Dragonfly (Object Research Systems, Montreal, QC, Canada). See the "X-ray data analysis" section in the Supplementary Materials for a detailed account of our processing pipeline.

Data Availability

The data used in this study can be found at, `https://datadryad.org/stash/dataset/doi:10.5061/dryad.z8w9ghxmw`.

Code Availability

The code used to analyze the X-ray data in this study can be found at `https://github.com/peleg-lab/honeycomb-adaptive-cell-size`.

References

1. Thomas D Seeley. *The wisdom of the hive: the social physiology of honey bee colonies*. Harvard University Press, 2009.

2. Paul Siefert, Nastasya Buling, and Bernd Grünewald. Honey bee behaviours within the hive: Insights from long-term video analysis. *Plos one*, 16(3):1–14, 2021.

3. Thomas C Hales. The honeycomb conjecture. *Discrete & Computational Geometry*, 25(1):1–22, 2001.

4. Forrest H Kaatz, Adhemar Bultheel, and Takeshi Egami. Order parameters from image analysis: a honeycomb example. *Naturwissenschaften*, 95(11):1033, 2008.

5. Tim Räz. On the application of the honeycomb conjecture to the bee's honeycomb. *Philosophia Mathematica*, 21(3):351–360, 2013.

6. John Hunter. Viii. observations on bees. *Philosophical Transactions of the Royal Society of London*, (82):128–195, 1792.

7. Charles Darwin. *On the origin of species, 1859*. London: John Murray, 1859.

8. DA Thompson. *On growth and form*. Cambridge : University Press ; New York, 1945.

9. Darcy Wentworth Thompson and D'Arcy W Thompson. *On growth and form*, volume 2. Cambridge university press Cambridge, 1942.

10. HR Hepburn. Comb construction by the african honeybee, apis mellifera adansonii. *Journal of the Entomological Society of Southern Africa*, 46(1):87–101, 1983.

11. Thomas D Seeley. *The lives of bees: the untold story of the honey bee in the wild*. Princeton University Press, 2019.

12. Peter R Marting, Benjamin Koger, and Michael L Smith. Manipulating nest architecture reveals three-dimensional building strategies and colony resilience in honeybees. *Proceedings of the Royal Society B*, 290(1998):20222565, 2023.

13. HR Hepburn and LA Whiffler. Construction defects define pattern and method in comb building by honeybees. *Apidologie*, 22(4):381–388, 1991.

14. TD Seeley and RA Morse. The nest of the honey bee (apis mellifera l.). *Insectes Sociaux*, 23(4):495–512, 1976.

15. Ming-Xian Yang, Ken Tan, Sarah E Radloff, Mananya Phiancharoen, and H Randall Hepburn. Comb construction in mixed-species colonies of honeybees, apis cerana and apis mellifera. *Journal of Experimental Biology*, 213(10):1659–1664, 2010.

16. Michael L Smith, Nils Napp, and Kirstin H Petersen. Imperfect comb construction reveals the architectural abilities of honeybees. *Proceedings of the National Academy of Sciences*, 118(31), 2021.

17. Golnar Gharooni Fard, Daisy Zhang, Francisco López Jiménez, and Orit Peleg. Crystallography of honeycomb formation under geometric frustration. *Proceedings of the National Academy of Sciences*, 119(48):e2205043119, 2022.

18. Shunhua Yang, Shangkao Deng, Haiou Kuang, Danyin Zhou, Xueyang Gong, and Kun Dong. Evaluating and comparing the natural cell structure and dimensions of honey bee comb cells of chinese bee, apis cerana cerana (hymenoptera: Apidae) and italian bee, apis mellifera ligustica (hymenoptera: Apidae). *Journal of Insect Science*, 21(4):1, 2021.

19. Shunhua Yang, Qingxin Meng, Wenzheng Zhao, Jianming Wang, Yiqiu Liu, Xueyang Gong, and Kun Dong. Cell orientation characteristics of the natural combs of honey bee colonies. *Plos one*, 17(2):e0263249, 2022.

20. Shivani Krishna, Apoorva Gopinath, and Somendra M Bhattacharjee. Ordering and topological defects in social wasps' nests. *Scientific Reports*, 12(1):12901, 2022.

21. Michael L Smith, Kevin J Loope, Bajaree Chuttong, Jana Dobelmann, James C Makinson, Tatsuya Saga, Kirstin H Petersen, and Nils Napp. Honey bees and social wasps reach convergent architectural solutions to nest-building problems. *PLoS Biology*, 21(7):e3002211, 2023.

22. Robert L Jeanne. The adaptiveness of social wasp nest architecture. *The quarterly review of biology*, 50(3):267–287, 1975.

23. John W Wenzel. Evolution of nest architecture. *The social biology of wasps*, pages 480–519, 1991.

24. Rahul Franklin, Sridhar Niverty, Brock A Harpur, and Nikhilesh Chawla. Unraveling the mechanisms of the apis mellifera honeycomb construction by 4d x-ray microscopy. *Advanced Materials*, 34(42):2202361, 2022.

25. HR Hepburn, CWW Pirk, and O Duangphakdee. Honeybee nests. *Composition, structure, function Springer*, 978(3):642–8, 2014.

26. Michael L Smith, Madeleine M Ostwald, and Thomas D Seeley. Adaptive tuning of an extended phenotype: honeybees seasonally shift their honey storage to optimize male production. *Animal Behaviour*, 103:29–33, 2015.

27. Scott Camazine. Self-organizing pattern formation on the combs of honey bee colonies. *Behavioral ecology and sociobiology*, 28:61–76, 1991.

28. H Randall Hepburn. *Honeybees and wax: an experimental natural history*. Springer Science & Business Media, 2012.

29. Robert Oeder and Dietrich Schwabe. The upward tilt of honeycomb cells increases the carrying capacity of the comb and is not to prevent the outflow of honey. *Apidologie*, 52:174–185, 2021.

30. Gabriele Von Oelsen and Eva Rademacher. Untersuchungen zum bauverhalten der honigbiene (apis mellifica). *Apidologie*, 10(2):175–209, 1979.

31. H Martin and M Lindauer. Sinnesphysiologische leistungen beim wabenbau der honigbiene. *Zeitschrift für vergleichende Physiologie*, 53:372–404, 1966.

32. Karl Müllenhoff. *Ueber die entstehung der bienenzellen*. Emil Strauss, 1883.

33. Guy Theraulaz and Eric Bonabeau. A brief history of stigmergy. *Artificial life*, 5(2):97–116, 1999.

34. Vince Gallo and Lars Chittka. Stigmergy versus behavioral flexibility and planning in honeybee comb construction. *Proceedings of the National Academy of Sciences*, 118(33):e2111310118, 2021.

35. François Huber. *Nouvelles observations sur les abeilles*, volume 1. JJ Paschoud, 1814.

36. Vincent Gallo and Lars Chittka. Cognitive aspects of comb-building in the honeybee? *Frontiers in psychology*, 9:900, 2018.

37. Thomas D Seeley. Adaptive significance of the age polyethism schedule in honeybee colonies. *Behavioral ecology and sociobiology*, 11:287–293, 1982.

38. EM Kindle. Some factors affecting the development of mud-cracks. *The journal of Geology*, 25(2):135–144, 1917.

39. Wai Peng Lee and Alexander F Routh. Why do drying films crack? *Langmuir*, 20(23):9885–9888, 2004.

40. Jianfeng Zang, Xuanhe Zhao, Yanping Cao, and John W Hutchinson. Localized ridge wrinkling of stiff films on compliant substrates. *Journal of the Mechanics and Physics of Solids*, 60(7):1265–1279, 2012.

41. Rashed Al-Rashed, Francisco López Jiménez, Joel Marthelot, and Pedro M Reis. Buckling patterns in biaxially pre-stretched bilayer shells: Wrinkles, creases, folds and fracture-like ridges. *Soft Matter*, 13(43):7969–7978, 2017.

Acknowledgments

O.P. and F.L.J. acknowledge funding from the University of Colorado Boulder RIO Seed Grant Program, and NSF grant 2210628. We also acknowledge funding from the University of Colorado Boulder, BioFrontiers Institute (internal funds). Any opinion, findings and conclusions or recommendations expressed in this material are those of the authors(s) and do not reflect the views of the

NSF. XRM data collection and parts of the analyses were performed at MIMIC facility, at CU Boulder (RRID:SCR_019307). We thank Dan Larremore for suggestions and help with data processing, Seneca Kristjonsdottir and Christopher Borke for bee management, Adrian Gestos for XRM training and trouble shooting, Paul Bontempo, Richard Terille, Anna Rahn, and Anna Simone for their assistance with data collection and organization.

Author Contributions

G.G., F.L.J. and O.P. designed research; G.G. performed research; G.G., C.K.P., F.L.J. and O.P. contributed new reagents/analytic tools; G.G. and C.K.P. analyzed data; G.G., C.K.P., F.L.J. and O.P. wrote the paper.

Competing Interests

The authors declare that they have no competing interests.

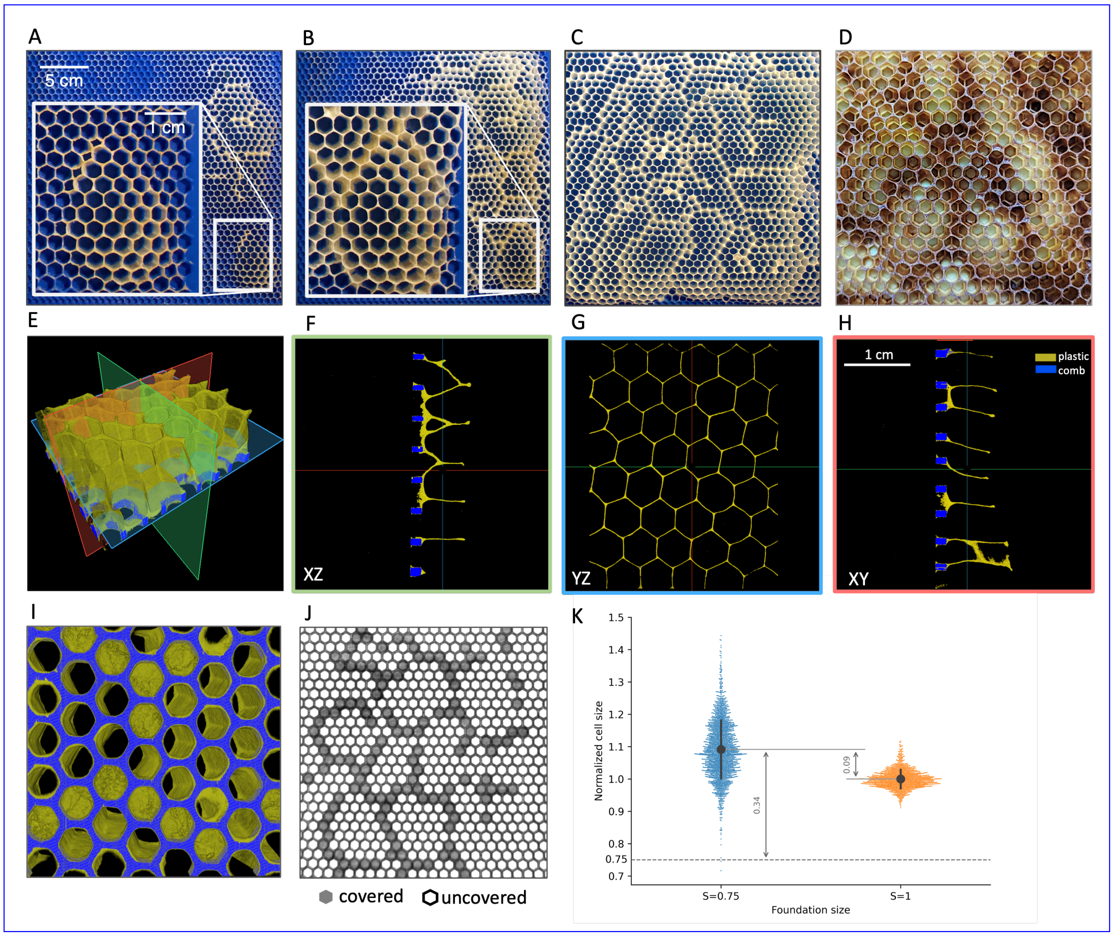

Figure 2: A) The 3D-printed frame of cells with $S = 0.75$ thinly coated with beeswax after 3 days of being in the hive. The inset shows the ~~zoomed in~~ zoomed-in section of the constructed comb highlighting cells with tilted walls. B) The same frame after a week. The inset shows the same~~zoomed in~~, but zoomed-in section ~~as~~ shown in panel A, demonstrating how the adjacent cells with tilted walls will be merged once they are fully constructed, effectively concealing the smaller cell below on the foundation. C) The same frame after ~~10~~ 20 days~~, and~~. D) ~~after 20 days~~A section of the honeycomb built on one of the non-experimental frames shows merging cells. E) 3D visualization of a ~~$5\ cm \times 5\ cm$~~ section of the comb intersected by three planes. ~~Three~~ F–H) The XZ, YZ, and XY planes shown in green, blue and red~~are used to create cross-sections of the comb structure, shown in F-H)~~. ~~In all of these slices the~~ The comb is shown in dark yellow while the plastic is ~~shown~~ in blue. I) The bottom view of the 3D reconstructed volume showing the cells that are covered ~~with comb~~ and ~~the~~ uncovered cells. J) 2D visualization of a larger section of the same sample with ~~uncovered cells in white and~~ covered cells highlighted in gray. K) Comparison of the ~~probability density function of~~ normalized cell ~~sizes~~ size distributions of cells built on foundations with $S = 0.75$ ~~, shown~~ in blue ~~, with~~ and cells built on the reference ~~cells, shown~~ frames with $S = 1$ in orange. The gray circles within each distribution indicate the mean for the distributions and the gray errorbars depict the standard deviations. The deviation in the mean cell size of cells built on $S = 0.75$ foundations is shown relative to both the reference cell size and the printed $S = 0.75$ cell size.

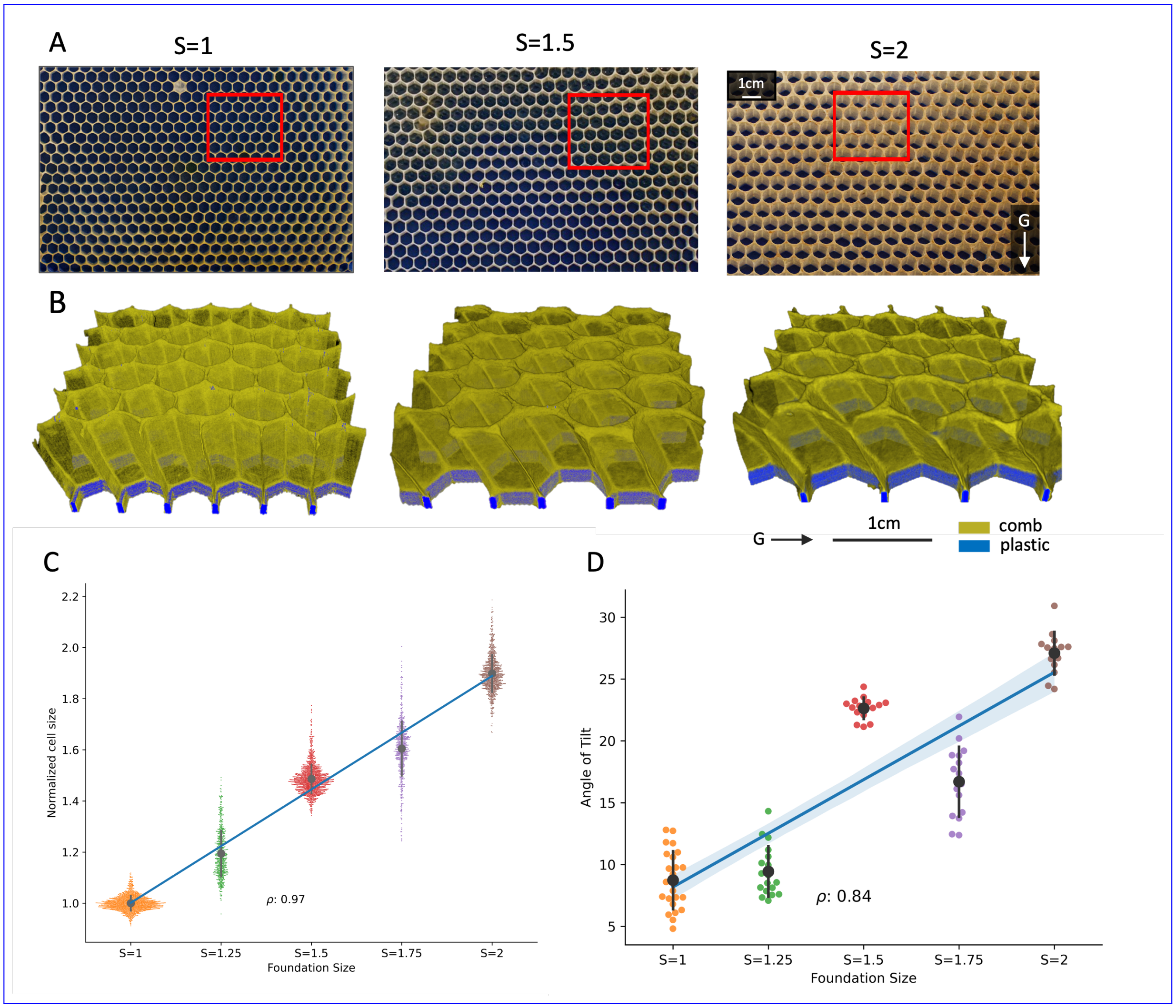

Figure 3: Results of the comb built on samples with $1 \leq S \leq 2$ foundation. A) The 2D images of the frame with $S = 1, 1.5$, and 2. B) The 3D reconstruction of the sections of the comb, highlighted with a red box in panel A, using X-ray data, segmented to show comb in dark yellow and the plastic in blue. C) Distribution of the ~~final~~ normalized cell sizes ~~for the comb~~ (C) and angles of tilt (D) of honeycomb cells (vertical axis) built on the frames with foundation size $1 \leq S \leq 2$ (horizontal axis), colored by foundation size. ~~The color shows~~ Wider spread in the ~~value~~ swarm of ~~S,~~ points indicates a higher number of data points at that normalized cell size. The gray circle within a swarm indicates the mean for that distribution with the gray error bars depicting the standard deviation. The blue lines in (C) and (D) indicate linear least-squares regression lines fit through the ~~vertical dashed line marks~~ data, while the ~~average cell size of natural drone brood cells~~shaded blue region around them illustrate the 95% confidence interval for the regression estimate.

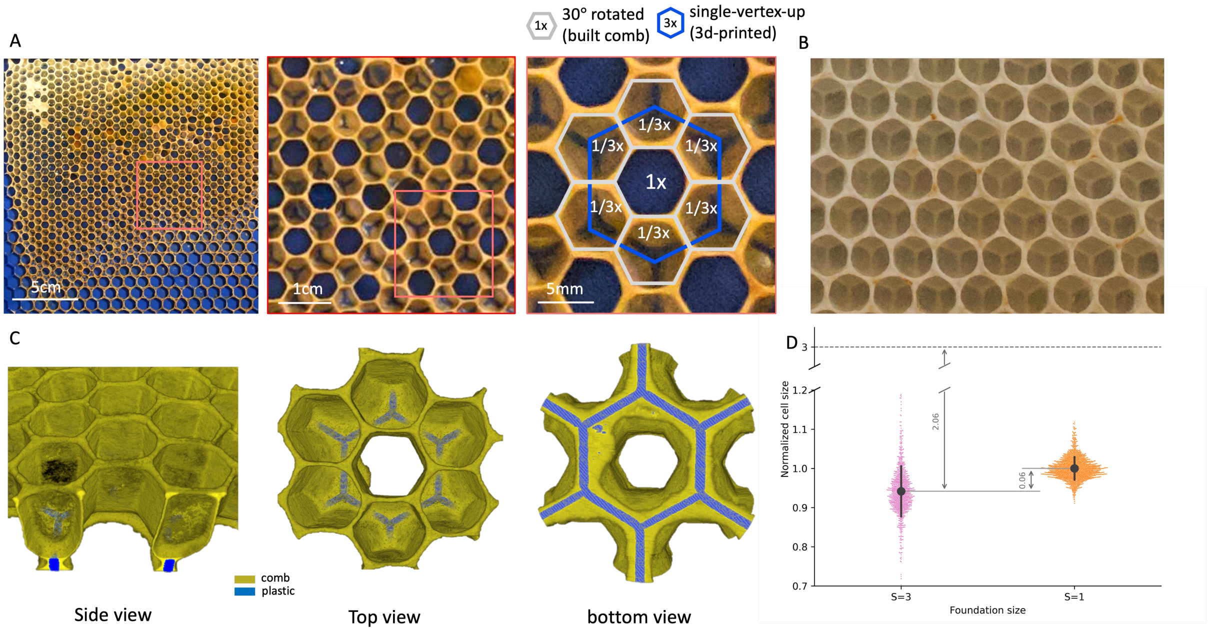

Figure 4: Results of the comb built on a sample with $S = 3$ foundation. A) Image of the comb built on the frame with $S = 3$, followed by the zoomed in view of the area highlighted with a red square. B) ~~Comparison~~ A piece of ~~the distribution of the final cell sizes built on foundations with $S = 3$ in yellow, with the ones built on the reference frame with $S = 1$ in gray~~natural two-sided honeycomb. C) Three views of the 3D reconstruction focusing on one ~~given~~ printed hexagon and the surrounding cells using X-ray data, showing the comb in dark yellow and the plastic in blue. D) Comparison of the normalized cell size distributions of cells built on foundations with $S = 3$ in pink and cells built on the reference frames with $S = 1$ in orange. The gray circles within the distributions indicate the mean for the distributions and the gray errorbars depict the standard deviations. The deviation in the mean cell size of cells built on $S = 3$ foundations is shown relative to both the reference cell and the printed $S = 3$ cell. Note that a section of the vertical axis has been truncated for better visibility of the necessary details.
