# Supplementary material for: Honeybees adapt to a range of comb cell sizes by merging, tilting, and layering their construction
Source: PLoS Biol. 2025 Aug 26;23(8):e3003253. doi: 10.1371/journal.pbio.3003253 (PMC12380279; doi:10.1371/journal.pbio.3003253)
Supplement: Attachment — Submitted filename: Response to PLOS Bio - with diff.pdf [file pbio.3003253.s015.pdf]

Response to Reviewers' comments for the manuscript
(PBIOLOGY-D-24-01668R2)

"Adaptive Cell Size, Merging, Tilting, and Layering in Honeybee Comb Construction"

Golnar Gharooni-Fard, Chethan Kavaraganahalli Prasanna,
Orit Peleg, and Francisco López Jiménez

We believe we have addressed all comments from the reviewers, in addition to other small changes to simplify notation and fix typos. Please refer to the "diff. version" appended at the end of this document for a detailed account of all the changes, corrections, and additions to the manuscript. The original text that was modified/deleted is struck-through (in red), and the revised/new text is underlined (in blue).

Response to Reviewer #1

Reviewer #1: General assessment
The authors have substantially improved the manuscript. The Introduction now situates the work firmly within the literature on stigmergic self organisation and collective construction, and the Discussion highlights the relevance for both evolutionary biology and bio inspired engineering. Most textual and citation issues raised previously have been resolved. Importantly, the authors augmented the Results section with quantitative analyses and confidence intervals.
Nevertheless, the statistical treatment still falls short of current best practice. Cell level observations arising from multiple frames and colonies are analysed with single level t and z tests that ignore non independence. Analysis of these data via mixed effects models would be recommended to obtain valid standard errors and to quantify colony to colony variability. Effect sizes should then be reported via estimated marginal means or contrasts with 95 % CIs.
With the mixed effects re analysis in place—together with minor editorial polishing—the manuscript will in my view meet the standards of PLOS Biology. I therefore recommend minor revision..

We thank the reviewers for carefully reading the paper, identifying the novelty of the work, and for the positive feedback. We have addressed each of the questions and comments below.

Specific comments

1. **Statistics**

- For every response variable analysed at cell level (covered cell proportion, cell area, tilt angle, etc.), it would be recommended to re fit a GLMM/LMM that includes colony (or colony × frame) as a random intercept and, where relevant, an observation level random effect to account for over dispersion. Subsequently, fixed effect estimates & 95 % CIs should be reported, alongside likelihood ratio or Wald p values. The packages that I recommended earlier (lme4, mclogit, emmeans) will handle this.

We appreciate the recommendation to employ mixed effects models and fully recognize the importance of accounting for hierarchical structure and potential non-independence in biological data. However, due to the nature of our experimental design and logistical constraints during data collection, we are unable to reliably attribute construction behavior to specific colonies, since we sometimes had to relocate frames between colonies (more details are provided later in “Section **2. Clarify replication structure**”). In particular, applying a generalized linear or linear mixed-effects model with colony as a random effect would not be possible as we did not keep track of the colonies that were involved in building the honeycomb on a particular frame. For these reasons, we chose simpler statistical comparisons at the cell level and included within-treatment variation estimates, while refraining from colony-based inferences.

Nonetheless, we appreciate the suggestion and agree that future experiments would benefit from a design that enables colony-level replication and statistical modeling. In particular, a larger-scale operation with more hives and fewer frame relocations would allow us to test for inter-colony variation in building strategies–something we are keen to pursue in follow-up work.

Although we are not able to model colony-level random effects, here are some of our results from using mixed models to model frame-level random effects. However, given the lack of added value these tests provided, we decided not to include them in the manuscript text.

1. *Covered cell proportion for S=0.75*
   For the covered cell proportion in the case of *S=0.75*, we do see some variation in proportions across the three frames that we have data for in this mode of construction.

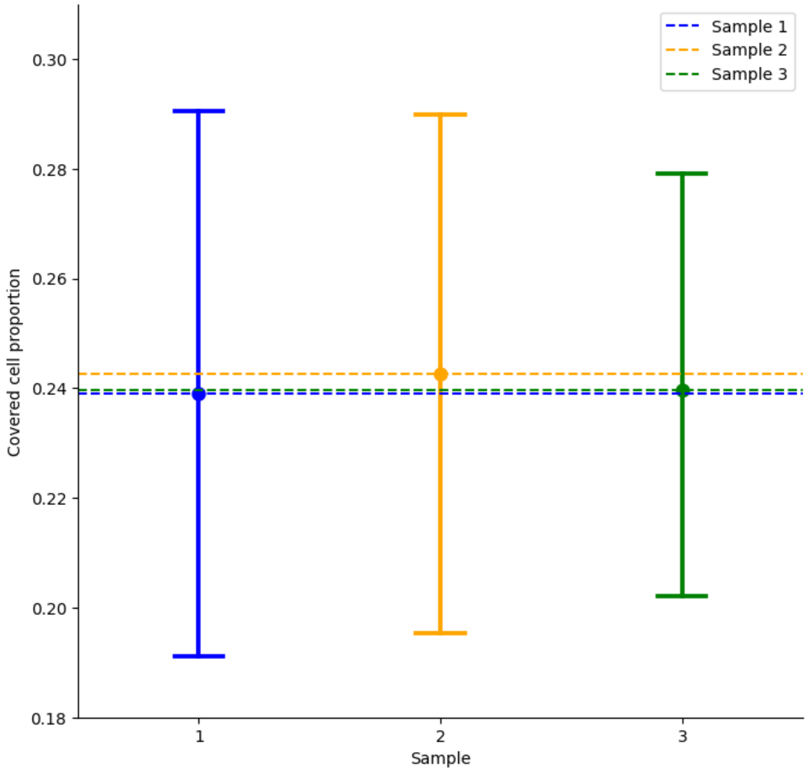

Considering frame-level random effects in this case can therefore help us get better estimates of standard errors. To do so, we can fit a logistic GLMM that has a binary response (*covered* or *not covered*) with a fixed-effect intercept that estimates the covered cell proportion and a random intercept that accounts for frame-level random effects.

```
glmer('Covered ~ 1 + (1|Sample)', data=o75_covered_cells_cell_level_df,
family="binomial")
```

However, we only have three replicates (frames) and the model is unable to reliably estimate the random-effect variance across frames, resulting in a *singular fit* error. Moreover, the proportion of covered cells across frames is very similar, leading to a near zero variance for the random-effect in our model, suggesting that a simpler fixed-effect model might be sufficient to describe our data. The results from the model are:

Formula: glmer(Covered~1+(1|Sample))

Family: *binomial (link: default)*
Number of observations: *1090*
Confidence intervals: *parametric*

Log-likelihood: *-601*
AIC: *1206* | BIC: *1216*
Residual error: *1.0*

Random Effects:		Estimate	CI-low	CI-high	SE	Z-stat	df	p	
Sample-sd	*(Intercept)*	0.000							
**Fixed Effects:**									
	(Intercept)	−1.151	−1.290	−1.012	0.071	−16.233	inf	<.001	***

2. *Built cell-size comparison between S=0.75 and. S=1*

The mean normalized cell size for *S=0.75* and *S=1* cells and the variance in their distribution, taking into consideration frame-level random effects in each construction mode, are estimated by the linear mixed model, fit separately for *S=0.75* and *S=1*:

```
lmer('NormalizedCellSize ~ 1 + (1|Sample)', data=df)
```

where, the fixed-effect intercept models the mean normalized cell-size for that construction mode and the random intercept models the frame-level random effects. *Sample* in the model refers to *Frame*. The results of the model are shown below:

For *S=1*:

Formula: lmer(NormalizedCellSize~1+(1|Sample))

Number of observations: *1364*
Confidence intervals: *parametric*

Log-likelihood: *3057*
AIC: *-6108* | BIC: *-6093*
Residual error: *0.025*

Random Effects:		Estimate	SE	CI-low	CI-high	T-stat	DF	p	
Sample-sd	*(Intercept)*	0.026							
Residual-sd	*Observation*	0.025							
**Fixed Effects:**									
	(Intercept)	1.010	0.013	0.969	1.051	78.700	2.978	<.001	***

For *S=0.75*:

Formula: lmer(NormalizedCellSize~1+(1|Sample))

Number of observations: *2178*
Confidence intervals: *parametric*

Log-likelihood: *2211*
AIC: *-4417* | BIC: *-4400*
Residual error: *0.087*

**Random Effects:**		**Estimate**	**SE**	**CI-low**	**CI-high**	**T-stat**	**DF**	**p**	
Sample-sd	*(Intercept)*	0.027							
Residual-sd	*Observation*	0.087							
**Fixed Effects:**									
	(Intercept)	1.094	0.014	1.051	1.138	79.983	2.984	<.001	***

Here, we see that the residual variance for cell-size, after having accounted for frame-level random effects within construction mode, is greater for built cells in *S=0.75* compared to *S=1*, showing that the distribution of normalized cell-sizes is wider for *S=0.75* than *S=1*. From these two models, although we see that the estimated fixed-effect mean normalized cell-size for *S=0.75* (1.094) is greater than *S=1* (1.01), the confidence intervals overlap, requiring a model that pools the *S=0.75* and *S=1* data to check whether the differences in their mean is significant. We build the pooled model as follows:

```
model = lmer("NormalizedCellSize ~ PrintedCellSize + (1|Sample)",
data=pl.concat([df_pl_1x, df_pl_o75x]))
```

where, the fixed-effect intercept estimates the mean normalized cell-size for *S=1* and the fixed-effect slope models how different the mean normalized cell-size for *S=0.75* is relative to *S=1*, taking into consideration the frame-level random effects within each construction mode. To clarify, PrintedCellSize is a categorical variable. The results of the model are shown below:

Formula: lmer(NormalizedCellSize~PrintedCellSize+(1|Sample))

Number of observations: *3542*
Confidence intervals: *parametric*

Log-likelihood: *4360*
AIC: *-8713* | BIC: *-8689*
Residual error: *0.07*

**Random Effects:**		Estimate	SE	CI-low	CI-high	T-stat	DF	p
Sample-sd	*(Intercept)*	0.026						

Residual-sd	*Observation*	0.070							
**Fixed Effects:**									
	(Intercept)	1.009	0.013	0.977	1.042	76.493	5.948	<.001	***
	PrintedCellSizeS=0.75	0.085	0.019	0.039	0.131	4.577	5.854	0.004023	**

This model shows that the mean normalized cell-size of *S=0.75* cells is indeed significantly larger than that of *S=1* cells, taking frame-level random effects within construction mode into consideration. However, we cannot measure the random effects in the cell-size difference (slope) itself, because we do not have crossed replicates across the *S=0.75* and *S=1* construction modes.

3. *Built cell-size comparison between S=3 and. S=1*

Similarly, the following results show that the mean normalized cell-size of *S=3* cells are smaller than *S=1* cells and their distribution is wider than that of *S=1* cells, taking into consideration frame-level random effects within each of these construction modes. Again, we cannot comment on the random effects in the mean cell-size difference (slope) because we lack the data to do this.

For *S=3*:

Formula: lmer(NormalizedCellSize~1+(1|Sample))

Number of observations: *982*
Confidence intervals: *parametric*

Log-likelihood: *1311*
AIC: *-2616* | BIC: *-2601*
Residual error: *0.063*

**Random Effects:**		Estimate	SE	CI-low	CI-high	T-stat	DF	p	
Sample-sd	*(Intercept)*	0.012							
Residual-sd	*Observation*	0.063							
**Fixed Effects:**									
	(Intercept)	0.942	0.008	0.911	0.973	122.259	2.165	<.001	***

Linear mixed-model to model the difference in the normalized cell-size of the *S=3* built cells from the *S=1* built cells, taking into consideration frame-level random effects within each construction mode:

Formula: lmer(NormalizedCellSize~PrintedCellSize+(1|Sample))

Number of observations: *2346*
Confidence intervals: *parametric*

Log-likelihood: *3912*
AIC: *-7816* | BIC: *-7793*
Residual error: *0.045*

**Random Effects:**		Estimate	SE	CI-low	CI-high	T-stat	DF	p	
Sample-sd	*(Intercept)*	0.021							
Residual-sd	*Observation*	0.045							

**Fixed Effects:**									
	(Intercept)	1.010	0.011	0.982	1.037	95.139	4.859	<.001	***
	PrintedCellSize S=3	−0.068	0.016	−0.110	−0.026	−4.171	4.898	0.009127	**

4. Cell-size trends

   The distribution of built cell sizes for each printed cell-size is shown by sample (a.k.a. replicate) below:

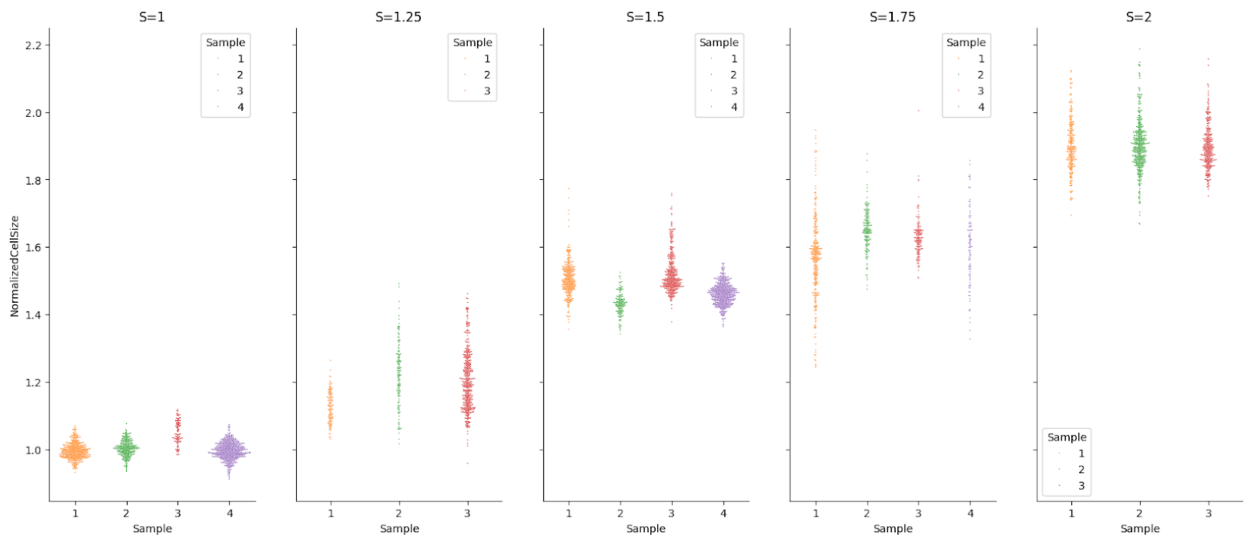

Taking into consideration the frame-level random effects within each construction mode, we model cell-size trends using the following linear mixed model:

```
model = lmer("NormalizedCellSize ~ PrintedCellSize + (1|Sample)", data=df_cell_size_trend)
```

where, we model the trend (slope) in the normalized built cell-size against the printed cell-size, with a random intercept modeling the frame-level random effects within construction modes. The results of this model are shown below:

Formula: lmer(NormalizedCellSize~PrintedCellSize+(1|Sample))

Number of observations: *5113*
Confidence intervals: *parametric*

Log-likelihood: *6749*
AIC: *-13490* | BIC: *-13464*
Residual error: *0.064*

**Random Effects:**		Estimate	SE	CI-low	CI-high	T-stat	DF	p	
Sample-sd	*(Intercept)*	0.048							
Residual-sd	*Observation*	0.064							
**Fixed Effects:**									
	(Intercept)	0.126	0.050	0.021	0.232	2.550	15.948	0.02144	*
	PrintedCellSize	0.874	0.032	0.805	0.943	26.915	15.944	<.001	***

We see a significantly positive slope of 0.874, indicating that an increase in one unit in the printed cell-size corresponds to an increase in 0.874 units in the built cell-size. However, we note that our experiments do not have a replication structure that allows us to estimate crossed random effects in the cell-size trend. As a result, we are only able to model frame-level random effects within each construction mode but not the random effects in the cell-size trends across different construction modes.

5. Angle of tilt trends

For angle data, we do not have multiple replicates per printed cell-size, and as a result we are unable to model random-effects even at the frame-level for angle of tilt trends.

2. Clarify replication structure

Specify the number of biological replicates (colonies) per treatment and how technical replicates (frames) are nested within colonies.

During the course of the experiment, frames were routinely moved between colonies due to practical considerations in our small-scale apiary. These included factors, such as colony growth, queen loss, swarming, disease, or changes in brood status. Because the experimental 3D-printed frames took one to two months to be fully built, it was often necessary to relocate frames to stronger or more active colonies to ensure construction was completed during the short honeybee season in Colorado. As a result, the final comb built on a given frame was often the product of multiple colonies over time. Given these constraints, our study was not designed to capture colony-specific behavior. Our main objective was to examine how honeybee groups – as a whole and across multiple colonies – handle variation in cell size, and whether consistent building modes emerge regardless of specific colony identity. Indeed, we find that bees from different colonies robustly adopt the same construction strategy across varied foundations. While we understand the value of colony-level analysis, our current dataset does not support assigning behavioral outcomes to specific colonies or quantifying colony-to-colony variability in a meaningful way.

In the Methods section, we included that each treatment was represented by at least three frames. We have now described the replication structure in the revised Methods section to clarify the nature of our data collection,

> *"During the season, we often move the experimental frames between the colonies, so we do not control the construction behavior at the colony level."*

Therefore, the level of replication in our analysis is at the frame level, not at the colony level.

3. Figures & tables

> **- Update Figs 2-4 to display model based estimates (mean ± 95 % CI) in addition to raw data.**
> **Ensure Table 1 lists mixed model estimates, not simple means.**

As explained before, we do not believe that our experiments allow us to perform a mixed model estimate that provides additional insight, and we are worried that introducing each panel as a sample could result in misleading results. While some frames are built by a single colony, others could have been built sequentially by more than one. As such, we believe that it is best to not change our analysis in this study. At the same time, we intend to track the connection between colonies and frames in our future research, with the goal of having a one-to-one relationship.

4. Terminology & editing
- Define XRM on first appearance.
- Maintain "printed" vs "built" cell terminology consistently.
- Verify that all supplementary figures cited in the text are present.

Response: We double-checked the paper for these points and they have all been verified. In particular, Fig.S1-S3 are referenced in the SI text section and Fig.S4-S7 are referenced in the main text of the paper.

> **5. Data & code**
> **Data and code availability statements are appreciated; please ensure that the GitHub repository also reproduces the statistical (ideally mixed model) analyses.**

The data is all available in Dryad, and all plots now indicate the data used.

The code has now been preserved in Zenodo, which avoids changes after publication. The code includes all tools used during our analysis.

Response to Reviewer #2

> **I have carefully reviewed the authors' responses to my comments and the corresponding revisions made to the manuscript. I am pleased to see that the authors have addressed all of my suggestions thoroughly and thoughtfully.**
>
> **The comprehensive revisions made by the authors in response to my and the other reviewers' comments have substantially improved the quality of the manuscript, which now presents a more cohesive, accurate, and convincing account of this interesting research on honeybee adaptive hive construction strategies.**
>
> **I am therefore satisfied that this manuscript is now suitable for publication in PLOS Biology.**

We thank the reviewer for the positive feedback and for the endorsement of our manuscript for publication in PLOS Biolgy.

Response to Reviewer #3

I have just a brief view on this paper. I appreciate the work the authors have put in to improve the accessibility of their paper and I think they have done a pretty good job with that. However, in so doing, they have shown that they are not able to describe the importance of their work: "Our experimental results extend our understanding of honeybees' remarkable comb-building adaptability, providing detailed evidence of their diverse construction techniques when faced with varying foundation cell sizes. Our findings align with and strengthen previous research by introducing more variety of construction modes in response to specific environmental conditions."

And the last line is still: "These future directions aim to enhance our comprehension of the intricate dynamics underlying comb construction and functionality of these adaptive structures.".

Response: Based on the reviewer's comments, we attempt to situate our findings within the broader context (literature) of animal architecture which can strengthen the relevance and accessibility of our work. In particular, we have made the following revisions:

1. Broader Framing in the Introduction:

   We revised the opening paragraphs of the Introduction to reference key examples of animal-built structures beyond honeybees, such as bird nests, termite mounds, and spider webs. This establishes a more comparative framework and underscores the relevance of our work to general principles of collective construction.

   "The ability of animals to construct nests that are well-adapted to their environment is crucial for their survival and reproduction. Across diverse taxa, from birds meticulously weaving nests [1] to termites engineering vast mounds [2, 3], we observe remarkable flexibility in building strategies in response to both internal needs and external environmental factors [4, 5]. This adaptive construction often involves optimizing resource use (e.g., caddisfly larvae selecting locally abundant materials for their cases [6]), improving functional performance against environmental challenges (e.g., termite mound thermoregulation using diurnal temperature oscillations [7]), and employing modular or iterative building processes and repairs (e.g., weaver bird communal nest structures [8, 9], or ants that alter their nest structure to slow the spread of diseases during colony expansions [10])."

2. Comparative Discussion:

We expanded the Discussion section to include a paragraph that explicitly compares the features of honeycomb construction to other collective or individual architectural behaviors in animals. This includes similarities in environmental adaptability, local rule-following, and emergent pattern formation.

"Our work highlights how honeybees, like many other architecturally proficient animals, do not follow a fixed blueprint to build their nest. Instead, they employ a surprisingly diverse repertoire of construction modes and adjustments in response to varying environmental conditions such as changes in foundation cell sizes. This collective flexibility enables them to generate adaptive solutions to local problems, contributing to the overall resilience and functionality of the colony's 'superorganismal' structure. The range of construction modes we documented in response to specific environmental conditions (i.e., 3D-printed cell size) indicates a sophisticated level of problem-solving and plasticity that parallels adaptive building in ants, spiders, termites, weaver birds, etc."

"In particular, the merging, tilting, and layering adaptations we observed represent strategies to preserve structural integrity and functional cell geometry under constraints–similar to how spiders adjust web tension in response to wind [46, 47], or how termites reinforce sections of their mound to effectively flush out CO2 and ventilate the nest [48]."

3. More Detailed Future Work

We expanded on the Future Work paragraph in the Discussion section to give more explicit examples of how our work can inspire future research.

"These findings not only deepen our knowledge of honeybee ingenuity but also contribute to a broader understanding of how complex, functional structures can emerge from adaptive behavioral responses. Our work envisions honeycombs not just as static structures, but as dynamically adapted solutions to a variety of environmental challenges."

"Beyond structural considerations, future research should aim to examine the underlying behavioral algorithms and sensory cues driving these adaptive construction choices in honeybees, potentially drawing parallels with decision-making processes in other animal builders. Investigating the response of bees to foundations with lattices introducing some added disorder or defective patterns such as non-hexagonal cells could further reveal how they interpret and resolve them. Comparative studies across social insects and other animal builders could uncover convergent evolutionary pathways for adaptive construction behaviors, enhancing our understanding of the complex dynamics and functional significance of these remarkable living structures within a broader ecological and evolutionary context."

We hope these additions make the manuscript more inclusive of and relevant to the broader literature on animal architecture, while still maintaining our main goal of pattern detection and analysis within the honeycomb structures.

Response to Reviewer #4

> **I understand most of my comments can not be addressed without extensive new experiments. This is completely justifiable. As said in my previous review I think the paper is interesting and deserves to be published.**

We thank the reviewer for their positive comments and understanding of our limitations, particularly due to the seasonal nature of our experiments. The suggestions will be taken into account into future experiments, so we thank their help in improving our future work.

Honeybees adapt to a range of comb cell size by merging, tilting and layering their construction

Golnar Gharooni-Fard[1], Chethan Kavaraganahalli Prasanna[1], Orit Peleg~~[1,2,3,4,5,6*]~~[1–3*], Francisco López Jiménez~~[7*]~~[4*]

1 Department of Computer Science, University of Colorado Boulder, CO, USA.
2 ~~Department~~ Departments of Physics, ~~University of Colorado Boulder, CO, USA.~~ ~~**3** Department of~~ Applied Math, ~~University of Colorado Boulder, CO, USA.~~ ~~**4** Department of Applied Math, University of Colorado Boulder, CO, USA.~~ ~~**5** Department of~~ and Ecology and Evolutionary Biology, University of Colorado Boulder, CO, USA.
~~6~~3 Santa Fe Institute, NM, USA.
~~7~~4 Department of Aerospace Engineering, University of Colorado, Boulder, CO, USA.

* Orit.Peleg@colorado.edu
* Francisco.LopezJimenez@colorado.edu

Abstract

Honeybees are renowned for their skills in building intricate and adaptive hives that display notable variation in cell size. However, the extent of their adaptability in constructing honeycombs with varied cell sizes has not been thoroughly investigated. We use 3D-printing and X-ray microscopy to quantify honeybees' capacity in adjusting the comb to different initial conditions. ~~Using the average area of natural worker cells as a reference, our~~ Our findings suggest three distinct ~~construction modes when faced with foundations of varying cell sizes~~ comb construction modes in response to foundations with varying sizes of 3D-printed cells. For smaller foundations, bees occasionally merge adjacent cells to compensate for the reduced space. However, for larger cell sizes, the hive uses adaptive strategies ~~like~~ such as tilting for foundations with cells up to twice the reference size and layering for cells that are three times larger than the reference cell. Our findings shed light on honeybees adaptive comb construction ~~modes~~abilities, significant for the biology of self-organized collective behavior, as well as for bio-inspired engineered systems.

Introduction

The ability of animals to construct nests that are well-adapted to their environment is crucial for their survival and reproduction. Across diverse taxa, from birds meticulously weaving nests [2] to termites engineering vast mounds [3, 4], we observe remarkable flexibility in building strategies in response to both internal needs and external environmental factors [5, 6]. This adaptive construction often involves optimizing resource use (*e.g.*, caddisfly larvae selecting locally abundant materials for their cases [7]), improving functional performance against environmental challenges (*e.g.*, termite mound thermoregulation using diurnal temperature oscillations [8]), and employing modular or iterative building processes and repairs (*e.g.*, weaver bird communal nest structures [9, 10], or ants that alter their nest structure to slow the spread of diseases during colony expansions [11]).

Honeybee nests are the emergent outcome of the collaborative efforts of thousands of individual bees. These structures play a vital role in the survival and functioning of a bee colony

by providing storage space for nectar and pollen, a nursery for brood development, and a structurally stable environment for the various interactions among the nestmates [12, 13]. The hexagonal shape of honeycomb cells, built with a near-optimal minimization of the wax-to-storage space ratio [14, 15, 16], has fascinated scientists for millennia ~~[17, 18, 19]~~ [17, 18, 20]. This optimization is necessary because wax production incurs a high energy cost [20]. In particular, to secrete just 1 *lb* (454 *g*) of wax, honeybees need to consume about 8 *lbs* (3.8 *kg*) of honey [21].

Feral bees have been shown to construct their nests on a range of surfaces such as tree branches and pre-existing cavities, which do not necessarily allow for regular hexagonal lattices [22]. Furthermore, comb building is a distributed process with no global blueprint or a leader for bees to follow. Instead, workers often initiate constructing the comb simultaneously from multiple locations within the hive [23]. To achieve a coherent structure, separate pieces of comb need to be merged and workers must reconcile the differing geometries of cell sizes at the boundaries (notably in terms of size and alignment), while conforming to the available space and resources [24]. The resulting structure inevitably contains non-regular hexagons and topological defects with various cell sizes, to adapt to environmental constraints [23, 25, 26, 27].

Adjusting the shape, size, and arrangement of hexagons during honeycomb construction may serve multiple purposes, often dictated by the environment or colony's requirements. Previous studies across various honeybee species have shown the precise design of their nest, optimized to maximize storage capacity and brood space [21, 28, 29]. These adaptations occur as the needs of the colony fluctuate, requiring more or less space for the rearing of the brood or the storage of honey over time [23]. ~~It is worth noting that honeybees are not the only species that adjust their nest structure based on external changes in the environment and colony requirements.~~ Social wasps have also been shown to use architectural solutions to build their nests, similar to honeybees [30, 31]. ~~The~~ For instance, the combs of paper wasps exhibit remarkable adaptability, with structural variations influenced by factors such as nest maturation, overall size, and cell arrangement [32, 33, 30].

More than four decades ago, H. R. Hepburn [21] studied the comb construction by African Honeybees *Apis melifera adansonii*, providing them with sheets of beeswax foundations having a range of cell sizes from 170 to 1022 $cells/dm^2$ (between 1 to 6 times the average size of the natural honeycomb cell). Using two-dimensional (2D) images of the built comb in the experimental frames, the study reported various construction solutions and measured the working tolerances of the comb with variable cell sizes. Yet, honeycomb represents a three-dimensional(3D) structure, and the details and intricacies of it cannot be fully discerned and quantified using ~~two-dimensional~~ 2D images. While the specific behavioral rules of individual bees during comb building remain unclear, revealing the 3D structure of the comb built under various initial conditions will be a crucial step in linking the local behavior to the global building patterns. For instance, the 3D progression of the nest building process, shown in a recent study by Marting et al [34], reveals that bees prioritize building a well-connected nest of parallel combs that remains structurally and functionally robust, even when faced with experimental disruptions. Such findings show the importance of accounting for 3D comb structure and provide valuable insights into the adaptive and resilient nature of the honeybee superorganism.

In this work, we study the adaptability of the comb construction by European honeybees *Apis melifera* L, under different initial conditions, using 3D visualization. We leverage 3D-printing to design repeatable experiments with precisely controlled and carefully quantified cell areas as foundations. By exploring the responses of honeybees to various initial conditions (using foundations with different cell sizes), our objective is to exhibit the range of construction modes bees use to build their nest, as well as the complexity of these structures and their sensitivity to initial conditions. Our findings corroborate and expand upon prior research by delineating the distinct construction modes employed by bees in response to specific cell sizes. To highlight the details of each construction mode, we use X-ray microscopy (XRM) to quantify

the properties and visualize these intricate structures. In a recent study, Franklin et al [35] have also used XRM technology to describe a step-by-step process by which honeycomb is constructed in natural settings, which sheds light on the structure of a natural honeycomb cell as it is being built. Their findings contribute to understanding the nest construction mechanism by showing how individual cells grow directly from the hexagonal pattern on the comb's spine, enhancing structural stability through staggered lattice patterns and strategic material deposition. Our work examines how foundations with different cell properties and arrangement give rise to distinct patterns and how these patterns can be interpreted in relation to the initial conditions on which they are built.

To distinguish various foundations, we define the cell size variable (S) as a multiple of the inner area of the average honeybee worker cell, that is derived from the length of a regular hexagon, which we measured to be 5.4 mm on a free comb built without a foundation. Fig. 1A shows a schematic of the shapes of the cells built on the plastic base with $S = 1$, equal to the size of an average worker cell. Fig. 1B shows two snapshots of a 3D-printed frame with $S = 1$ at the start and end of the comb building process. Fig. 1C shows the 3D-visualization of the structure of the comb using X-ray data from a section of the frame shown in panel B. The details of how we process the raw X-ray data to generate 3D-visualizations of the comb structure are described in the Methods Section. Using $S = 1$ as our control, we supply the hives with custom 3D-printed frames with regular hexagonal patterns of various cell sizes, ranging from half to four times the size of a reference cell (*i.e.*, $0.5 \leq S \leq 4$). Our goal in changing the ~~foundation's~~ 3D-printed cell sizes is to examine the bees' capacity to adjust the comb structure to the given foundation. Fig. 1D provides an overview of our results, highlighting different construction modes observed on our 3D-printed frames. It includes photographs of the obtained structures followed by schematics of adaptive strategies used for honeycomb construction with varying cell sizes.

Fig 1. Overview of results for different values of S. A) Schematic of the honeycomb cells built on a control frame with $S = 1$. B) Two snapshots of the initial frame with the plastic, 3D-printed foundation along with the same frame after 20 days. The printed cell size on this frame is $S = 1$. C) XRM-based 3D-visualization of a section of the frame with $S = 1$, segmented to highlight the comb in dark yellow and the plastic in blue. The direction of gravity is indicated by the arrow labeled *G*. D) Overview of the different modes of honeycomb construction on 3D-printed frames with varying cell sizes. The top row shows the building mode in relation to the cell sizes on each foundation, displayed beneath them. Images of all the built frames used in this work are organized by 3D-printed cell size and are available in 2D_image.zip. The subsequent row presents images of the ~~constructed~~ built honeycomb on the foundations, accompanied by a zoomed-in view beneath. The bottom row illustrates schematics depicting adaptive strategies for honeycomb construction with differing cell sizes.

Results

We begin this section by discussing the different construction modes identified in our experimental data, followed by a more detailed description and visualization of the overall structure of the comb in each mode. We start our exploration by reducing the area of the 3D-printed cells on the foundations to half of the average worker cell size (*i.e.* $S = 0.5$). Our findings suggest that when faced with such small foundations, bees do not follow the patterns or use the printed cells in any other form for comb building. The leftmost column in Fig. 1 D shows that bees fill most of the small printed cells with wax and build new hexagons on a mostly flat foundation. On the other end, increasing the size of the printed cells to four times greater than the size of the average worker cell ($S = 4$) did not produce any consistent patterns (see the rightmost column in Fig. 1 D. However, within the range of $0.75 \leq S \leq 3$, we identify three

different building modes—merging, tilting, and layering—relative to the size of the printed foundation. The intricate properties of each construction mode, along with 3D-visualization using XRM, are presented in the following sections.

Merging

When faced with foundations where cell sizes are 0.75 times smaller than the size of an average worker cell ($S = 0.75$), bees adapt the comb structure and use the foundation with an unexpectedly intricate approach. Fig. 2 A–D illustrates the progression of comb construction on one such experimental frame over a 20-day period. There is an apparent pattern at work from the early stages of comb construction, indicating the swarms' recognition of and adaptation to this new cell size pattern. As shown in the insets of Fig. 2 A–B, even though most of the drawn hexagons start from the plastic edges on the printed foundation, some cell walls are built with an angle that eventually merges with the walls of adjacent cells built with a reverse tilt. This pattern repeats until the entire frame is covered with hexagonal cells. The term "merging" describes the occasional combination of several small cells to create the necessary space to construct natural worker-size hexagons.

Fig 2. Results of the comb built on samples with $S = 0.75$ foundation.. A) The 3D-printed frame of cells with $S = 0.75$ thinly coated with beeswax after 3 days of being in the hive. The inset shows the zoomed-in section of the constructed comb highlighting cells with tilted walls. B) The same frame after a week. The inset shows the same, but zoomed-in section shown in panel A, demonstrating how the adjacent cells with tilted walls will be merged once they are fully ~~constructed~~built, effectively concealing the smaller cell below on the foundation. C) The same frame after 20 days. D) A section of the honeycomb built on one of the non-experimental frames shows merging cells. E) 3D visualization of a section of the comb intersected by three planes. F–H) The XZ, YZ, and XY planes shown in green, blue and red. The comb is shown in dark yellow while the plastic is in blue. I) The bottom view of the 3D reconstructed volume showing the cells that are covered and uncovered cells. J) 2D visualization of a larger section of the same sample with covered cells highlighted in gray. K) Comparison of the normalized cell size distributions of cells built on foundations with $S = 0.75$ in blue and cells built on the reference frames with $S = 1$ in orange. The gray circles within each distribution indicate the mean for the distributions and the gray errorbars depict the standard deviations. The deviation in the mean cell size of cells built on $S = 0.75$ foundations is shown relative to both the reference cell size and the printed $S = 0.75$ cell size. The raw data for (K) is available in cell_size_data.zip under S_1 and S_o75.

Fig. 2 C shows the frame after comb construction is complete, but prior to filling of cells with honey or brood, at which point X-ray imaging is conducted. We find that the merging behavior during honeycomb construction is not specific to our experimental frames with smaller printed foundations. A similar pattern can sometimes be observed on the comb built on some of the commercially available frames with worker-size foundations (equivalent to our 3D-printed frames with $S = 1$). Fig. 2 D shows an image of the comb built on one of the commercial frames inside our hives using the merging mode. This adjustment may be made to meet the colony's specific needs for constructing larger cells, such as those used for raising drones. The series of images in Fig. 2 E–H demonstrate the X-ray results. In particular, Fig. 2 E and Supplementary Movie S1 illustrates a 3D-reconstruction of a 5 cm $\times$ 5 cm section of the frame in Fig. 2 C, highlighting plastic in blue and comb in dark yellow. To better visualize the merging process, the cross-sections of the X-ray are presented in Fig. 2F-H, using planes containing two of the Cartesian axes, shown in green (XZ), blue (YZ), and red (XY) in Fig. 2 E. For example, the XZ slice in Fig. 2 F shows how the comb material is used to join adjacent plastic edges beneath the cells.

Remarkably, while some of the printed cells are filled with wax, the top view of the comb structure, shown in Fig. 2 G, still features a regular hexagonal grid with cell sizes that are comparable to the average worker cell size. Fig. 2 K depicts the distribution of normalized cell size for cells built on frames with $S = 0.75$ in blue, and the same for reference cells ($S = 1$) in orange. The normalized cell size is computed by dividing the built cell areas by the mean reference cell area. For better interpretability, we will use normalized cell sizes in future discussions, unless otherwise stated. As depicted in Fig. 2 K, the mean normalized cell size built on foundations with $S = 0.75$ is 0.09 units larger than the mean reference cell size, but 0.34 units larger than the area of the corresponding printed cells $S = 0.75$. This suggests that on foundations with $S = 0.75$, bees are building honeycomb similar to the typical cell size that is closer to the reference cells than to those on the printed foundation. Moreover, apart from being slightly larger than the reference cell size ($\mu_{\text{S=0.75}} = 1.09$, $\mu_{S=1} = 1$, $t = 44.0385, \text{P} < 10^{-5}$ for a one-tailed two-sample Welch's unequal variances t-test), the variation in cell size for these cells is considerably wider ($\sigma_{\text{S=0.75}} = 0.09$, $\sigma_{S=1} = 0.03$, $W = 971.173, \text{P} < 10^{-5}$ for the Levene test to compare variances), suggesting less consistency in the cell sizes of the comb built on the smaller foundations. The higher standard deviation could be attributed to the discrepancy between the printed and desired cell sizes and the inherent randomness of the merging process. See Table S1 in the ~~Supplementary Materials~~ Supporting Information section for more details on the number of samples in each category, the individual sample size and statistics.

Additionally, in the bottom view of the sample in Fig. 2 I, it can be observed that some of the printed cells are entirely covered with comb. This coverage potentially aims to provide additional space for the initiation of the hexagonal lattice, with $S \approx 1$, which, as noted earlier, is what bees construct on the $S = 0.75$ foundations by merging cells. The distribution of covered printed cells on a larger section of the frame can be viewed in Fig. 2 J, with covered cells shown in gray. If we hypothesize that bees compensate for the 25% reduction in space per cell (relative to their average worker cell size) by combining cells in our 3D-printed foundations with $S = 0.75$, we would expect them to cover 25% ($h_0 = 0.25$) of the cells in the printed foundation to build a hexagonal lattice with $S \approx 1$. Interestingly, all individual experimental samples, as well as the pooled data, fail to reject our null hypothesis ($h_0 = 0.25$) under a two-tailed one-sample z-test for proportions at a significance level $\alpha = 0.05$ (when pooled together: $h_{\text{pooled}} = 0.24, \text{z} = -0.744, \text{P} = 0.457$). For details about the individual replicates, see Table S2 in the ~~Supplementary Materials~~ Supporting Information section. This suggests that bees employ the merging strategy to effectively transform a slightly smaller hexagonal grid ($S = 0.75$) into their commonly used size ($S = 1$), and the behavior has been consistent across our ~~samples~~experiments.

Tilting

The next mode of construction corresponds to the case with the printed foundation of cells with $1 \leq S \leq 2$, where bees use the provided patterns but increase the tilt angle of the built cells as the cell size increases. Fig. 3 A presents a series of 2D images of the comb built on frames with $S = 1, 1.5$, and 2. The corresponding 3D reconstructions of the comb built on these frames are presented in Fig. 3 B, which are the result of scanning sections of the combs highlighted with red boxes in Fig. 3 A. Tracing the plastic edges shown in blue and the comb in dark yellow across the three samples displayed in Fig. 3 B reveals a consistent behavior: bees follow the printed foundations, regardless of increasing cell sizes.

The distribution of the size of the cells built on frames with foundations with $S = 1, 1.5, 2$ is shown in Fig. 3 C, with their detailed statistics shown in Table. 1. A linear least-squares regression line that fits the cell size data, using normalized cell size as the response and the foundation size as the predictor, has a positive slope that is statistically significant ($\beta = 0.89, P < 10^{-5}$), demonstrating an increasing trend of built cell sizes with the foundation size. Moreover, Fig. 3 C suggests that this increasing trend in cell sizes is monotonic, with the

strength of the trend highlighted by a high correlation coefficient (Pearson's correlation coefficient $\rho = 0.97, P < 10^{-5}$), indicating a strong positive relationship between the foundation and the final cell size. This provides strong evidence of the bees' ability to effectively utilize the printed foundations by adjusting honeycomb cell shapes and sizes in response to structural changes. According to our observations (which are consistent with previous findings [*36, 24, 37, 38*]), these larger cells (built on foundations with $1 < S \leq 2$) are not suitable for raising workers, which are normally raised in cells built with $S \approx 1$. Therefore, these larger cells are either used for raising drones or storing honey. See Fig. S4 for some examples of the usages of these cells in our experimental frames. For context, the average drone cell size [*39*] when normalized is equal to $S = 1.61$.

Fig 3. Results of the comb built on samples with $1 \leq S \leq 2$ foundation. A) The 2D images of the frame with $S = 1, 1.5$, and 2. B) The 3D reconstruction of the sections of the comb, highlighted with a red box in panel A, using X-ray data, segmented to show comb in dark yellow and the plastic in blue. C) Distribution of the normalized cell sizes (C) and angles of tilt (D) of honeycomb cells (vertical axis) built on the frames with foundation size $1 \leq S \leq 2$ (horizontal axis), colored by foundation size. Wider spread in the swarm of points indicates a higher number of data points at that normalized cell size. The gray circle within a swarm indicates the mean for that distribution with the gray error bars depicting the standard deviation. The blue lines in (C) and (D) indicate linear least-squares regression lines fit through the data, while the shaded blue region around them illustrate the 95% confidence interval for the regression estimate. The raw data for (C) is available in cell_size_data.zip under S_1, S_1o25, S_1o5, S_1o75, and S_2. The data underlying (D) is available in angle_data.zip.

As shown in Fig. 3D, we find that bees significantly increase the tilt of cells as the foundation sizes increase. Previous studies exploring the natural tilt of honeycomb cells have indicated that a standard worker cell tilts up to 13° [*39, 25, 40*]. It has also been established that the direction of this tilt is influenced by gravity: bees consistently tilt cells upward [*41, 42, 28*]. In our experiments, we note that cells on the control frames with $S = 1$ are tilted, on average, by $8.74°$ against the direction of gravity. For comparison, we computed the average tilt angle of natural drone cells to be $20°$ (see Fig S5 for more information). The tilt of the drone comb is measured manually using images of natural drone comb built on a flat surface, such as Fig. S5. The mean angle of tilts, their 95% confidence intervals and standard deviations for each foundation size $1 \leq S \leq 2$ are shown in Table 1.

Fig. 3D shows the tilt distribution of the comb cells built on various foundation sizes, along with a best-fit linear regression line. It is evident that there is a gradual increase in cell tilts with increasing values of S, with a linear least-squares regression line fit to the angle data (response: angle of tilt, predictor: foundation size) showing a statistically significant positive slope ($\beta = 17.36, P < 10^{-5}$). Although the linear positive trend is strong (Pearson's correlation coefficient: $\rho = 0.84, P < 10^{-5}$), this relationship is not strictly monotonic. In particular, cells built on foundations with $S = 1.75$ are, on average, less tilted compared to those with $S = 1.5$ (under a one-tailed two-sample Welch's unequal variances t-test, $t = -8.04, P < 10^{-5}$). This trend suggests that cell size is not the only influential parameter and the tilt may be influenced by various structural factors of the hive, such as cell position on the frame, usage, or content.

Previous studies have suggested various hypotheses to explain the natural tilt of honeycomb cells. Mullenhoff [*43*] posited that this tilt prevents honey outflow. However, a recent study by Oeder et al. [*40*] challenges this idea, proposing instead that the tilt redistributes weight onto the midwall, potentially increasing the comb's carrying capacity. Additionally, in our experiments, we find that the amount of tilt effectively reduces the cross-sectional area of the cell, when considered perpendicular to the walls. When we cut the cells of the sample built on foundations with $S = 2$ using a plane perpendicular to the walls at the top of the sample, we find that the cross-sectional area of the hexagons in that layer is, on average, 0.77 times smaller than the area of the printed hexagons at its bottom. In other words, the cross-sectional area is equivalent to

$S = 1.54$, a significant reduction compared to the size of the provided foundation $S = 2$. Moreover, these tilted cells also have a larger depth, defined along the direction of the walls, for a given total volume of the cell. In general, the angle of tilt could represent a compromise between maximizing volume, ensuring structural stability, and optimizing construction efficiency. See Supplementary Movies S2-6 for the 3D reconstruction of all the samples built with tilting cells.

Table 1. Mean and standard deviation of cell sizes and tilts measured across various printed cell sizes (S)

Foundation cell size	Normalized cell size			Tilt [degrees]		
	**Mean (μ)**	**Std. dev. (σ)**	**95% CIs**	**Mean (μ)**	**Std. dev. (σ)**	**95% CIs**
1	1	0.03	[1.00, 1.00]	8.74	2.34	[7.75, 9.73]
1.25	1.19	0.09	[1.19, 1.20]	9.45	2.03	[8.44, 10.46]
1.5	1.49	0.06	[1.48, 1.49]	22.64	0.86	[22.18, 23.10]
1.75	1.6	0.1	[1.60, 1.61]	16.71	2.82	[15.20, 18.21]
2	1.89	0.07	[1.89, 1.90]	27.1	1.72	[26.10, 28.09]

Layering

Lastly, we explore cells built on frames with areas three times larger than worker cells (*i.e.* $S = 3$), which leads to a new mode of construction; see Fig. 4. Unlike the previous case, bees do not adapt the provided pattern simply by constructing larger and more tilted cells. Instead, they use the vertices of 3D-printed cells as the foundation for a new layer of cells, forming a two-layer structure; see Fig. 4 A. This ability to build cells that are centered and supported by the edges of previously built cells reflects their natural behavior. Fig. 4 B shows a piece of a two-sided comb from a hive without foundation, where the front side is built upon the structural support of the pre-existing lattice on the back. The geometry of the 3D-printed ~~panels~~ frames in our experiments resulted in the emergence of an additional cell, located at the center of the printed hexagons. This cell extends to the base of the 3D-printed frame, merging with the printed hexagon. The positioning and structure of the cells are more clearly illustrated in the 3D view of the comb shown in Fig. 4 C, highlighting how the honeycomb cells (dark yellow) are built atop the plastic edges (blue).

Fig. 4 D depicts the distribution of normalized cell sizes of cells built on foundations with $S = 3$ and the reference cells ($S = 1$), with their means and standard deviations included. We find that the mean normalized cell size of cells built on foundations with $S = 3$ is 0.06 units smaller than the mean of the normalized reference cells, whereas these cells are 2.06 units smaller than the printed cells in the $S = 3$ frames, showing that cells built on foundations with $S = 3$ are much closer in size to reference worker cells than to their printed foundations. Typically, these cells are smaller than reference worker cells ($\mu_{\text{S=3}} = 0.94$, $\mu_{\text{S=1}} = 1$,

Fig 4. Results of the comb built on samples with $S = 3$ foundation. A) Image of the comb built on the frame with $S = 3$, followed by the zoomed in view of the area highlighted with a red square. B) A piece of natural two-sided honeycomb. C) Three views of the 3D reconstruction focusing on one printed hexagon and the surrounding cells using X-ray data, showing the comb in dark yellow and the plastic in blue. D) Comparison of the normalized cell size distributions of cells built on foundations with $S = 3$ in pink and cells built on the reference frames with $S = 1$ in orange. The gray circles within the distributions indicate the mean for the distributions and the gray errorbars depict the standard deviations. The deviation in the mean cell size of cells built on $S = 3$ foundations is shown relative to both the reference cell and the printed $S = 3$ cell. Note that a section of the vertical axis has been truncated for better visibility of the necessary details. The raw data for (D) is available in cell_size_data.zip under S_1, S_3.

$t = -26.67, \mathrm{P} < 10^{-5}$ for a one-tailed two-sample Welch's unequal variances t-test), while the variation in their normalized cell sizes is wider than that of the reference cell size distribution ($\sigma_{\mathrm{S=3}} = 0.06$, $\sigma_{\mathrm{S=1}} = 0.03$, $W = 285.59, \mathrm{P} < 10^{-5}$ for the Levene test to compare variances). The similarity in size of the cells built on $S = 3$ foundations to that of the reference cells suggests that the two-layer strategy employed by bees effectively transforms the printed foundation of $S = 3$, which is not suitable for any purpose in the hive, to $S = 1$, which is the most commonly used.

Overall, this symmetrical structure shown in in Fig. 4 A, is composed of six newly constructed built hexagons layered over the original foundation, forming another hexagon in its center. Consequently, as illustrated in the zoomed-in image in Fig. 4 A, each gray hexagon in the top layer derives approximately one-third of its area from the 3D-printed (blue) cell and the remaining two-thirds from the neighboring cells on which it is built. Another notable aspect of this structure is the change in lattice orientation during this geometric transformation. Although we printed the hexagons on the foundation with a single-vertex-up orientation, the honeycomb lattice constructed built by the bees exhibits a 30° rotated structure (as shown by the blue and gray hexagons in Fig. 4 A). While the former orientation is commonly used in commercially available foundations and it is the orientation most observed in naturally built comb, the latter has been found to be approximately 30% stronger structurally [*21*]. Further increase in the printed cell sizes (*e.g.* $S = 4$) did not produce any new patterns. We will discuss more details about all the tested patterns in the following section.

Discussion

The flexibility of comb building behavior in bees has been previously discussed as a result of stigmergy [*44*] combined with a highly rich behavioral repertoire owned by bees [*45*]. Our experimental results extend our understanding of honeybees' remarkable comb-building adaptability, providing detailed evidence of their diverse construction techniques when faced with varying work highlights how honeybees, like many other architecturally proficient animals, do not follow a fixed blueprint to build their nest. Instead, they employ a surprisingly diverse repertoire of construction modes and adjustments in response to varying environmental conditions such as changes in foundation cell sizes. Our findings align with and strengthen previous research by introducing more variety of construction modes This collective flexibility enables them to generate adaptive solutions to local problems, contributing to the overall resilience and functionality of the colony's 'superorganismal' structure. The range of construction modes we documented in response to specific environmental conditions (*i.e.*, 3D-printed cell size) indicates a sophisticated level of problem-solving and plasticity that parallels adaptive building in ants, spiders, termites, weaver birds, etc. The observed variety of solutions might suggest cognitive planning [*46, 47*]–where , wherein bees potentially maintain a mental template of the desired outcome and select appropriate building modes based on foundation geometry–our study primarily focuses on how bees demonstrate multiple effective strategies for managing geometric challenges related to cell size, while maintaining structural functionalitygeometry. However, addressing the cognitive aspects of forward planning at the colony level would require dedicated targeted experiments that are beyond the scope of this paper. Here, our primarily focus remains on how bees demonstrate multiple effective strategies for managing geometric challenges related to cell size. In particular, the merging, tilting, and layering adaptations we observed represent strategies to preserve structural integrity and functional cell geometry under constraints–similar to how spiders adjust web tension in response to wind [*48, 49*], or how termites reinforce sections of their mound to effectively flush out CO_2 and ventilate the nest [*50*].

Within the range of cell sizes we examined, which is We examined how bees respond to foundation cell sizes within the range of $0.5 \leq S \leq 4$. Notably, when $S = 0.5$, or $S = 4$, it became apparent that bees do not effectively utilize the patterns on the provided foundation

~~when $S = 0.5$, or $S = 4$~~use the printed foundation patterns. For $S = 0.5$, ~~the bees~~ they either disregard the printed pattern entirely or fill the small cells with wax (see Fig. 1D)~~. This allows~~ , enabling them to initiate ~~the~~ comb construction on a flat foundation. This behavior resembles the nesting practices of wild honeybees found in tree cavities with numerous small cracks and crevices [*51*]~~. In such natural settings, bees tend to create a flat and waterproof surface by using~~ , where they apply tree resins (propolis) to seal~~and coatthe tiny gaps and cracks, effectively preventing the growth of~~ , coat, and create a smooth, waterproof base that protects against mold and bacteria [*22*]. ~~In the case of~~ At the oposite extreme, when $S = 4$, ~~the bees are~~ bees are similarly unable to use the printed foundations to build a uniform structure. ~~Most of the comb built on this size foundation seem~~ Instead, their comb appears to be randomly constructed in all directions, sometimes growing outwards perpendicular to the frame (see Fig. 1D). ~~We have also collected data using foundations with other~~ For intermediate sizes such as $S = 2.5$ and $S = 3.5$~~. On these frames we observed an~~ , we observed inconsistent mix of tilted and layered hexagonal cells ~~,~~ (see Fig. S6 on the ~~Supplementary Materials~~ Supporting Information section for examples), suggesting more difficulty to generate uniform structures using a single mode of construction at these scales.

For the remaining foundations tested ($0.75 \leq S \leq 3$) our results show different mechanisms by which honeybees adapt regular hexagonal foundations of different sizes to respond to the imposed structural changes while satisfying ~~their~~ colony needs. In the cases ~~with~~ of $S = 0.75$ and $S = 3$, the ~~provided~~ 3D-printed cell sizes are quite different from those naturally used by bees, ~~so they need to adapt them to~~ requiring notable adjustments to build a new lattice. In the case of smaller cells, the size mismatch between both layers is resolved by effectively blocking some ~~of the~~ 3D-printed cells, ~~so that their space is distributed among their neighbor cells~~redistributing their space among adjacent ones. This can be understood as the discrete version of systems in which geometric mismatch between two layers is resolved through localization of deformation, from cracks in drying films or mud [*52, 53*] to ridges in pre-stretched elastic bilayers [*54, 55*], which result in similar patterns to those created by the blocked hexagonal cells. ~~In the case of~~ For $S = 3$, ~~the~~ bees use the plastic edges of the ~~provided frame as a foundation~~ printed frame as scaffolds to build a new ~~regular hexagonal lattice. The new hexagons are~~ layer of hexagonal lattice, centered on the edges and vertices of the ~~provided lattice , constructing a new layer of hexagonal lattice with~~ foundation. This reconstructed lattice has a cell size close to $S = 1$~~. This structure closely resembles the comb construction seen~~ , closely mirroring comb construction in African honeybees ~~on a foundation~~ documented in [*21*]. While Hepburn described this middle cell as a “false” cell ~~that is~~ not easily noticed in the completed comb, our ~~experiments reveal~~ observations suggest that the broad base of this central cell can serve as an additional storage space for honey and pollen. In ~~contrast, in~~ the range $1 \leq S \leq 2$, ~~the~~ bees extend the provided lattice, constructing larger cells ~~while increasing the tilt of the cells as their size increases.~~ as S increases. These larger cells are often more tilted, suggesting a structural or functional adaptation linked to increased volume. For a comprehensive comparison of the cell sizes ~~constructed~~ built on all provided foundations, please refer to Fig. S7 in the ~~supplementary materials section.~~ Supporting Information section. These findings not only deepen our knowledge of honeybee ingenuity but also contribute to a broader understanding of how complex, functional structures can emerge from adaptive behavioral responses. Our work envisions honeycombs not just as static structures, but as dynamically adapted solutions to a variety of environmental challenges.

In our previous work [*27*], we introduced a 2D model capable of replicating the patterns and defects in the comb structure arising from various geometric frustrations. ~~With~~ Building upon the identification of novel building modes, particularly the two-layered pattern in our current study, we are motivated to extend our 2D model into a 3D framework. This extension will enable us to delve deeper into understanding the potential costs and benefits associated with such structural adaptations, which could be leveraged in the design of novel lightweight structures conforming to complex irregular geometries. Furthermore, given the bees’

demonstrated capacity to construct and utilize large tilted cells, our future work involves rationalizing the relationship between cell size and angle, starting with the two reasons already proposed in the literature. With that objective in mind, we will examine the by examining the mechanical stability of the comb under larger tilt values, as well as heavily tilted cells and the fluid mechanics that govern honey outflow within these structures. Future work could also test the response of the

Beyond structural considerations, future research should aim to examine the underlying behavioral algorithms and sensory cues driving these adaptive construction choices in honeybees, potentially drawing parallels with decision-making processes in other animal builders. Investigating the response of bees to foundations with lattices introducing some added disorder or defective patterns such as non-hexagonal cells could further reveal how they interpret and resolve them. Comparative studies across social insects and other animal builders could uncover convergent evolutionary pathways for adaptive construction behaviors, enhancing our understanding of the complex dynamics and functional significance of these remarkable living structures within a broader ecological and evolutionary context. These future directions aim to enhance our comprehension of the intricate dynamics underlying comb construction and functionality of these adaptive structures .

Methods

To collect data about the structure of the comb, we establish a set of behavioral assays combined with X-ray imaging. The focus of this study is on the cell size ($0.5 \leq S \leq 4$) which is varied systematically using 3D-printed panels frames so that its effect can be isolated and quantified. The design work for preparing the 3D-printed frames is conducted using the SolidWorks 2019 CAD design software. All of our experiments are performed using colonies of European honeybees *Apis melifera* L. at the Peleg lab apiary in Boulder, Colorado. We use langstroth hives with 19 in (480 mm) frames, with $9\frac{1}{8}$ in (230 mm) depth, and $1\frac{3}{8}$ in (35 mm) width. Each frame consists of a pair of 3D-printed plates of the size 212×212 mm that are placed opposite to each other. For our control set with $S = 1$, each hexagonal element on the 3D-printed plates has a side-length of 2.7 mm and outer area of 18.94 mm^2 (including the cell walls). All the other configurations are designed relative to the area of our control set. To confirm the observed modes of construction, we collected at least three repetitions of samples for each cell size, S. See the Supplementary Data section of the Supplementary Materials for pictures of these samples. To account for at least three repetitions of each of the configurations under study, we scatter a total of 50 3D-printed experimental frames into ten honeybee colonies and fill the rest of the space in the hives with normal frames with full plastic foundations. In general, each colony has half 3D-printed frames and half normal frames. During the season, we often move the experimental frames between the colonies, so we do not control the construction behavior at the colony level.

To stimulate comb building, we apply a fine layer of natural beeswax to the printed foundation. The comb construction progress is monitored through regular photography sessions, capturing images of both sides of the experimental frames. To ensure minimal disruption during photography, we set up our photography station within the apiary, allowing us to quickly return the frames to their original locations inside the hives. Once the experimental frames are fully built, they are carefully extracted from the hives, and kept in the lab for further analysis. We obtain high-quality images of the comb using a Nikon DSLR camera and controlled lighting, and a black background. The camera is mounted on a fixed metal structure 2 m from the comb to avoid distortion at the edges of the image and to keep a constant pixel-to-millimeter relationship. At this distance and camera resolution, 1 mm = 15 pixels.

To further analyze the honeycomb structures obtained after the completion of the experiments, we used 3D X-ray microscopy (XRM) to scan 5 cm $\times$ 5 cm sections of the comb constructed on our experimental frames. This provides a series of high resolution cross-sections

of pieces of the comb that can be combined to generate a 3D virtual model. All tomography scans are performed using $\mu - CT$ ZEISS Xradia 520 Versa (Carl Zeiss X-ray Microscopy Inc, Pleasanton, CA, USA). The X-ray source is set to voltages in range 80-90 kV and power 7 W, while the detector camera is set with an exposure time of 2 seconds for each of the 2034 images captured by optical magnification 0.4. Image processing and segmentation on the X-ray data are conducted using Dragonfly (Object Research Systems, Montreal, QC, Canada). See the "X-ray ~~data analysis~~Data Analysis" section in the ~~Supplementary Materials~~ Supporting Information for a detailed account of our processing pipeline.

Data Availability

The data used in this study can be found ~~at,~~ in `https://datadryad.org/stash/dataset/doi:10.5061/dryad.z8w9ghxmw`.

Code Availability

The code used to analyze the X-ray data in this study can be found ~~at~~ in
`https://zenodo.org/records/15610301?preview=1&token=`
`eyJhbGciOiJIUzUxMiJ9.`
`eyJpZCI6Ijc2NDI4ZDBiLTVlNjgtNDMzNy05ZThlLWZlYzI2NzdmMDM1MiIsImRhdGEiO[illegible]`
`11CmVTvTV-EVjEKlqthSA5aaGXkcmmrrTguZcKaMwFeymP2DyEOxLpfjHCni--ht47StZ[illegible]`

~~**S1 Fig.** Bold the title sentence.Add descriptive text after the title of the item (optional).~~

Supporting information

S1 Text. Additional information on X-ray data analysis.

~~**S2**~~ **S1 Fig. Processing of X-ray tomography scans.** A) X-ray tomography data before processing: the 3D visualization followed by samples of three cross-sections along different orthogonal planes. B) Fraction of pixels with a given intensity in a tomography image showing a sharp peak corresponding to air pixels in the image. The vertical red-dashed line shows the threshold value computed to filter out air (background). C) Histogram thresholding to segment out plastic cell edges. The blue peak corresponds to plastic cell edges and the red-dashed line highlights the optimum threshold value to segment out plastic region. D) Final results after running the complete image processing pipeline, in which honeycomb (dark yellow) and plastic cell edges (blue) are segmented.

S2 Fig. Calculation of cell tilt angle. Angle of tilt calculation for individual honeycomb cells demonstrated on a sample with S = 2. A) ~~A sample~~ An image from the XZ slice, showing the two parallel planes that cross the sample perpendicular to the plastic edges. B) Identifying individual cells using connected component analysis on a cross-section of the sample with the planes shown in A. C) Results of automatic centroid detection on the 3D volume detecting cell centroids in blue (for the top plane) and red (for the lower plane). The angle of tilt for each cell is calculated using the value of the shift in the centroids for each individual cell.

S3 Fig. Honeycomb on commercial frame. Honeycomb built on one of the commercial frames inside our hives using the merging strategy. A section of comb is magnified to show the occasional combination of the plastic edges on the foundation to build larger cells on top of them.

S4 Fig. Samples with $S > 1$. Sample frames with larger cell size foundations are either used for raising drone brood or honey storage.

S5 Fig. Drone cells. Honeycomb containing drone brood built on the flat side of the experimental frames. This is used to compute the value of 20° as the natural tilt of the drone comb, with a standard deviation of 2.43° and 95% CI: [18.86, 21.14].

S6 Fig. Samples with $S = 2.5$ and $S = 3$. Samples of acquired data on frames with $S = 2.5$, and $S = 3.5$ show a combination of tilted or layered modes of building on random sections of the frames.

S7 Fig. Cell size distribution. Comparison of cell size distribution of honeycomb built on all of the ~~3d-printed~~ 3D-printed frames. The given cell sizes (S) are shown with different colors in the figure legend. The raw data used in this figure is available in cell_size_data.zip.

S1 Table. Cell size statistics across various printed cell sizes.

S2 Table. Covered cell statistics for $S = 0.75$. The data used for this table is available in s_o75_covered_cell_data.zip.

S3 Table. Description of ~~the supplementary movie files~~supplementary movie files.

S1 Movie. 3D scan of sample with $S = 0.75$

S2 Movie. 3D scan of sample with $S = 1$

S3 Movie. 3D scan of sample with $S = 1.25$

S4 Movie. 3D scan of sample with $S = 1.5$

S5 Movie. 3D scan of sample with $S = 1.75$

S6 Movie. 3D scan of sample with $S = 2$

S7 Movie. 3D scan of sample with $S = 3$

Acknowledgments

XRM data collection and parts of the analyses were performed at MIMIC facility, at CU Boulder (RRID:SCR_019307). We thank Dan Larremore for suggestions and help with data processing, Seneca Kristjonsdottir and Christopher Borke for bee management, Adrian Gestos for XRM training and trouble shooting, Paul Bontempo, Richard Terille, Anna Rahn, and Anna Simone for their assistance with data collection and organization.

Author Contributions

G.G., F.L.J. and O.P. designed research; G.G. performed research; G.G., C.K.P., F.L.J. and O.P. contributed new reagents/analytic tools; G.G. and C.K.P. analyzed data; G.G., C.K.P., F.L.J. and O.P. wrote the paper.

Competing Interests

The authors declare that they have no competing interests.

References

1. ~~Thomas D Seeley~~

2. Collias NE, Collias EC. Nest building and bird behavior. Princeton University Press; 2014.

3. Turner JS, et al. Architecture and morphogenesis in the mound of Macrotermes michaelseni (Sj~~The~~ östedt)(Isoptera: Termitidae, Macrotermitinae) in northern Namibia. Cimbebasia. 2000;16(January 2000):143–175.

4. Turner JS. The extended organism: the physiology of animal-built structures. Harvard University Press; 2009.

5. Medina I. The role of the environment in the evolution of nest shape in Australian passerines. Scientific Reports. 2019;9(1):5560.

6. Bates JM, Fidino M, Nowak-Boyd L, Strausberger BM, Schmidt KA, Whelan CJ. Climate change affects bird nesting phenology: Comparing contemporary field and historical museum nesting records. Journal of Animal Ecology. 2023;92(2):263–272.

7. Wiggins GB. Caddisflies: the underwater architects. University of Toronto Press; 2005.

8. King H, Ocko S, Mahadevan L. Termite mounds harness diurnal temperature oscillations for ventilation. Proceedings of the National Academy of Sciences. 2015;112(37):11589–11593.

9. Collias EC, Collias NE. The development of nest-building behavior in a weaverbird. The Auk. 1964;81(1):42–52.

10. Collias NE, Collias EC. Weaverbird nest aggregation and evolution of the compound nest. The Auk. 1977; p. 50–64.

11. Rajendran H, Weinberger R, Fonio E, Feinerman O. Colony demographics shape nest construction in ants. bioRxiv. 2024; p. 2024–07.

12. Seeley TD. The wisdom of the hive: the social physiology of honey bee colonies. Harvard University Press~~,~~ ; 2009.

13. ~~Paul Siefert , Nastasya Buling , and Bernd~~ Siefert P, Buling N, Grünewald B. Honey bee behaviours within the hive: Insights from long-term video analysis. ~~Plos one,~~ Plos one. 2021;16(3):1–14~~, 2021.~~ .

14. ~~Thomas C Hales~~ Hales TC. The honeycomb conjecture. Discrete & Computational Geometry~~,~~ . 2001;25(1):1–22~~, 2001.~~ .

15. ~~Forrest H Kaatz , Adhemar Bultheel , and Takeshi Egami~~ Kaatz FH, Bultheel A, Egami T. Order parameters from image analysis: a honeycomb example. ~~Naturwissenschaften,~~ Naturwissenschaften. 2008;95(11):~~1033, 2008.~~ 1033.

16. ~~Tim~~ Räz T. On the ~~application of the honeycomb conjecture to the bee's honeycomb~~Application of the Honeycomb Conjecture to the Bee's Honeycomb. ~~Philosophia Mathematica,~~ Philosophia Mathematica. 2013;21(3):351–360~~, 2013.~~ .

17. ~~John Hunter~~ Hunter J. ~~Viii. observations~~ VIII. Observations on bees. Philosophical Transactions of the Royal Society of London~~, (~~. 1792;82~~)~~:128–195~~, 1792.~~ .

18. ~~Charles Darwin~~ Darwin C. On the origin of species, ~~1859.~~ 1859. London: John Murray~~,~~ ; 1859.

19. ~~DA Thompson~~

20. Thompson DW. On growth and form~~. Cambridge : University Press ; New York, 1945.~~ ~~Darcy Wentworth Thompson and D'Arcy W Thompson.On growth and form, volume~~. vol. 2. Cambridge university press Cambridge~~,~~ ; 1942.

21. ~~HR Hepburn~~ Hepburn H. Comb construction by the ~~african honeybee, apis~~ African honeybee, Apis mellifera adansonii. Journal of the Entomological Society of Southern Africa~~,~~ . 1983;46(1):87–101~~, 1983.~~ .

22. ~~Thomas D Seeley~~ Seeley TD. The lives of bees: the untold story of the honey bee in the wild. Princeton University Press~~,~~ ; 2019.

23. ~~HR Hepburn and LA Whiffler~~ Hepburn H, Whiffler L. Construction defects define pattern and method in comb building by honeybees. ~~Apidologie,~~ Apidologie. 1991;22(4):381–388~~, 1991.~~ .

24. ~~TD Seeley and RA Morse~~ Seeley T, Morse R. The nest of the honey bee (~~apis mellifera l~~Apis mellifera L.). ~~Insectes Sociaux,~~ Insectes Sociaux. 1976;23(4):495–512~~, 1976.~~ .

25. ~~Ming-Xian Yang , Ken Tan , Sarah E Radloff , Mananya Phiancharoen , and H Randall Hepburn~~ Yang MX, Tan K, Radloff SE, Phiancharoen M, Hepburn HR. Comb construction in mixed-species colonies of honeybees, ~~apis cerana and apis~~ Apis cerana and Apis mellifera. Journal of Experimental Biology~~,~~ . 2010;213(10):1659–1664~~, 2010.~~ .

26. ~~Michael L Smith , Nils Napp , and Kirstin H Petersen~~ Smith ML, Napp N, Petersen KH. Imperfect comb construction reveals the architectural abilities of honeybees. Proceedings of the National Academy of Sciences~~,~~ . 2021;118(31)~~, 2021.~~ .

27. ~~Golnar Gharooni Fard , Daisy Zhang , Francisco López~~ Fard GG, Zhang D, Jiménez ~~, and Orit Peleg~~ FL, Peleg O. Crystallography of honeycomb formation under geometric frustration. Proceedings of the National Academy of Sciences~~,~~ . 2022;119(48):e2205043119~~, 2022.~~ .

28. ~~Shunhua Yang , Shangkao Deng , Haiou Kuang , Danyin Zhou , Xueyang Gong , and Kun Dong~~ Yang S, Deng S, Kuang H, Zhou D, Gong X, Dong K. Evaluating and comparing the natural cell structure and dimensions of honey bee comb cells of ~~chinese bee, apis~~ Chinese bee, Apis cerana cerana (~~hymenoptera~~Hymenoptera: Apidae) and ~~italian bee, apis~~ Italian bee, Apis mellifera ligustica (~~hymenoptera~~Hymenoptera: Apidae). Journal of Insect Science~~,~~ . 2021;21(4):~~1, 2021.~~ 1.

29. ~~Shunhua Yang , Qingxin Meng , Wenzheng Zhao , Jianming Wang , Yiqiu Liu , Xueyang Gong , and Kun Dong~~Yang S, Meng Q, Zhao W, Wang J, Liu Y, Gong X, et al. Cell orientation characteristics of the natural combs of honey bee colonies. ~~Plos one,~~ Plos one. 2022;17(2):e0263249~~, 2022.~~ .

30. ~~Shivani Krishna , Apoorva Gopinath , and Somendra M Bhattacharjee~~ Krishna S, Gopinath A, Bhattacharjee SM. Ordering and topological defects in social wasps' nests. ~~Scientific Reports,~~ Scientific Reports. 2022;12(1):~~12901, 2022.~~ 12901.

31. ~~Michael L Smith , Kevin J Loope , Bajaree Chuttong , Jana Dobelmann , James C Makinson , Tatsuya Saga , Kirstin H Petersen, and Nils Napp~~Smith ML, Loope KJ, Chuttong B, Dobelmann J, Makinson JC, Saga T, et al. Honey bees and social wasps reach convergent architectural solutions to nest-building problems. ~~PLoS Biology,~~ PLoS Biology. 2023;21(7):e3002211~~, 2023.~~.

32. ~~Robert L Jeanne~~ Jeanne RL. The adaptiveness of social wasp nest architecture. The quarterly review of biology~~,~~. 1975;50(3):267–287~~, 1975.~~.

33. ~~John W Wenzel~~ Wenzel JW. Evolution of nest architecture. The social biology of wasps~~, pages~~. 1991; p. 480–519~~, 1991.~~.

34. ~~Peter R Marting , Benjamin Koger , and Michael L Smith~~ Marting PR, Koger B, Smith ML. Manipulating nest architecture reveals three-dimensional building strategies and colony resilience in honeybees. Proceedings of the Royal Society B~~,~~. 2023;290(1998):~~20222565, 2023.~~ 20222565.

35. ~~Rahul Franklin , Sridhar Niverty , Brock A Harpur , and Nikhilesh Chawla~~ Franklin R, Niverty S, Harpur BA, Chawla N. Unraveling the ~~mechanisms of the apis mellifera honeycomb construction by 4d x-ray microscopy~~Mechanisms of the Apis mellifera Honeycomb Construction by 4D X-ray Microscopy. ~~Advanced Materials,~~ Advanced Materials. 2022;34(42):~~2202361, 2022.~~ 2202361.

36. ~~HR Hepburn , CWW Pirk , and O Duangphakdee~~ Hepburn H, Pirk C, Duangphakdee O. Honeybee nests. Composition, structure, function Springer~~,~~. 2014;978(3):642–8~~, 2014.~~.

37. ~~Michael L Smith , Madeleine M Ostwald , and Thomas D Seeley~~ Smith ML, Ostwald MM, Seeley TD. Adaptive tuning of an extended phenotype: honeybees seasonally shift their honey storage to optimize male production. ~~Animal Behaviour,~~ Animal Behaviour. 2015;103:29–33~~, 2015.~~.

38. ~~Scott Camazine~~ Camazine S. Self-organizing pattern formation on the combs of honey bee colonies. Behavioral ecology and sociobiology~~,~~. 1991;28:61–76~~, 1991.~~.

39. ~~H Randall Hepburn~~ Hepburn HR. Honeybees and wax: an experimental natural history. Springer Science & Business Media~~,~~; 2012.

40. ~~Robert Oeder and Dietrich Schwabe~~ Oeder R, Schwabe D. The upward tilt of honeycomb cells increases the carrying capacity of the comb and is not to prevent the outflow of honey. ~~Apidologie,~~ Apidologie. 2021;52:174–185~~, 2021.~~.

41. ~~Gabriele~~ Von Oelsen ~~and Eva Rademacher~~ G, Rademacher E. Untersuchungen zum ~~bauverhalten der honigbiene (apis~~ Bauverhalten der Honigbiene (Apis mellifica). ~~Apidologie,~~ Apidologie. 1979;10(2):175–209~~, 1979.~~.

42. ~~H Martin and M Lindauer~~ Martin H, Lindauer M. Sinnesphysiologische leistungen beim wabenbau der honigbiene. Zeitschrift für vergleichende Physiologie~~,~~. 1966;53:372–404~~, 1966.~~.

43. ~~Karl~~ Müllenhoff K. Ueber die entstehung der bienenzellen. Emil Strauss~~,~~; 1883.

44. ~~Guy Theraulaz and Eric Bonabeau~~ Theraulaz G, Bonabeau E. A brief history of stigmergy. ~~Artificial life,~~ Artificial life. 1999;5(2):97–116~~, 1999.~~.

45. ~~Vince Gallo and Lars Chittka~~ Gallo V, Chittka L. Stigmergy versus behavioral flexibility and planning in honeybee comb construction. Proceedings of the National Academy of Sciences~~,~~. 2021;118(33):e2111310118~~, 2021.~~.

46. ~~François Huber~~ Huber F. Nouvelles observations sur les abeilles~~, volume~~. vol. 1. JJ Paschoud~~,~~ ; 1814.

47. ~~Vincent Gallo and Lars Chittka~~ Gallo V, Chittka L. Cognitive aspects of comb-building in the honeybee? Frontiers in psychology~~,~~ . 2018;9:~~900, 2018.~~ 900.

48. Wu CC, Blamires SJ, Wu CL, Tso IM. Wind induces variations in spider web geometry and sticky spiral droplet volume. Journal of Experimental Biology. 2013;216(17):3342–3349.

49. Tew N, Hesselberg T. The effect of wind exposure on the web characteristics of a tetragnathid orb spider. Journal of Insect Behavior. 2017;30:273–286.

50. Ocko SA, Heyde A, Mahadevan L. Morphogenesis of termite mounds. Proceedings of the National Academy of Sciences. 2019;116(9):3379–3384.

51. ~~Thomas D Seeley~~ Seeley TD. Adaptive significance of the age polyethism schedule in honeybee colonies. Behavioral ecology and sociobiology~~,~~ . 1982;11:287–293~~, 1982.~~ .

52. ~~EM Kindle~~ Kindle E. Some factors affecting the development of mud-cracks. The journal of Geology~~,~~ . 1917;25(2):135–144~~, 1917.~~ .

53. ~~Wai Peng Lee and Alexander F Routh~~ Lee WP, Routh AF. Why do drying films crack? ~~Langmuir,~~ Langmuir. 2004;20(23):9885–9888~~, 2004.~~ .

54. ~~Jianfeng Zang , Xuanhe Zhao , Yanping Cao , and John W Hutchinson~~ Zang J, Zhao X, Cao Y, Hutchinson JW. Localized ridge wrinkling of stiff films on compliant substrates. Journal of the Mechanics and Physics of Solids~~,~~ . 2012;60(7):1265–1279~~, 2012.~~ .

55. ~~Rashed~~ Al-Rashed ~~, Francisco~~ R, López Jiménez ~~, Joel Marthelot , and Pedro M Reis~~ F, Marthelot J, Reis PM. Buckling patterns in biaxially pre-stretched bilayer shells: Wrinkles, creases, folds and fracture-like ridges. ~~Soft Matter,~~ Soft Matter. 2017;13(43):7969–7978~~, 2017.~~ .
